# Diverse Microbial Composition of Sourdoughs From Different Origins

**DOI:** 10.3389/fmicb.2020.01212

**Published:** 2020-07-15

**Authors:** Andrea Comasio, Marko Verce, Simon Van Kerrebroeck, Luc De Vuyst

**Affiliations:** Research Group of Industrial Microbiology and Food Biotechnology (IMDO), Faculty of Sciences and Bioengineering Sciences, Vrije Universiteit Brussel (VUB), Brussels, Belgium

**Keywords:** sourdough, metagenetics, metagenomics, lactic acid bacteria, acetic acid bacteria, yeasts, species diversity

## Abstract

Hundreds of sourdoughs have been investigated in the last decades. However, many studies used a culture-dependent and/or culture-independent microbiological approach [mainly based on denaturing gradient gel electrophoresis (DGGE) of PCR amplicons], seldomly combined with a metabolite target analysis, to characterize the microbial species communities of the sourdoughs examined. Moreover, attention was mainly paid on lactic acid bacteria (LAB) and yeast species. In the present study, distinct household-scale (including an artisan lambic brewery) and artisan bakery-scale backslopped sourdoughs (17 in total), obtained from different regions (Belgium, France, United Kingdom, and USA), were examined through a multiphasic approach, encompassing a culture-dependent analysis [targeting LAB, acetic acid bacteria (AAB), and yeasts], different culture-independent techniques [rRNA-PCR-DGGE, metagenetics, and metagenomics (four bakery sourdoughs)], and metabolite target analysis. It turned out that the microbial species diversity of the sourdoughs was influenced by the house microbiota of the producer. Further, when the producer made use of different flours, the sourdoughs harbored similar microbial communities, independent of the flour used. AAB were only present in the Belgian sourdoughs, which might again be related to the processing environment. *Fructilactobacillus sanfranciscensis* (formerly known as *Lactobacillus sanfranciscensis*) was the prevalent LAB species of the eight sourdoughs produced by two of the three bakeries of different countries analyzed. These sourdoughs were characterized by the presence of either *Saccharomyces cerevisiae* or *Kazachstania humilis*. Moreover, the presence of *Fl. sanfranciscensis* was positively correlated with the production of mannitol and negatively correlated with the presence of other LAB or AAB species. Sourdoughs produced in an artisan lambic brewery were characterized by the presence of the yeast species *Dekkera anomala* and *Pichia membranifaciens*. One household sourdough was characterized by the presence of uncommon species, such as *Pediococcus parvulus* and *Pichia fermentans*. Metagenomic sequencing allowed the detection of many more LAB and AAB species than the other methods applied, which opened new frontiers for the understanding of the microbial communities involved during sourdough production processes.

## Introduction

Sourdoughs are matrices of mainly cereal flour and water that are fermented by means of lactic acid bacteria (LAB; mainly heterofermentative LAB species) and yeasts (De Vuyst et al., 2016, 2017; Settanni, 2017; Van Kerrebroeck et al., 2017; Gobbetti et al., 2019). The LAB to yeast ratio is mostly 10:1 to 100:1 (Gobbetti, 1998; Hammes et al., 2005; De Vuyst et al., 2014, 2017). Not only cereal flours from wheat, rye, spelt, and barley but also flours from pseudocereals, legumes, and seeds are used (Coda et al., 2014; De Vuyst et al., 2014, 2017). Sourdough production is generally carried out through backslopping, whereby each backslopping step is characterized by a spontaneous fermentation of the flour-water mixture, thanks to the wild microorganisms present in the flour, other ingredients, or the environment; alternatively, it can be initiated with starter cultures (De Vuyst et al., 2014, 2017). Backslopped sourdoughs often harbor particular consortia of LAB and/or yeasts. For instance, San Francisco sourdough harbors a microbial consortium of *Fructilactobacillus sanfranciscensis* (formerly known as *Lactobacillus sanfranciscensis*; Zheng et al., 2020) and *Kazachstania humilis* (formerly *Candida humilis*) that is the result of a nutritional mutualism and thanks to mutual stress responses (Gänzle et al., 1998; Vogel, 2015; Jacques et al., 2016; De Vuyst et al., 2017). However, other consortia of maltose-positive LAB and maltose-negative yeasts occur (e.g., *Fl. sanfranciscensis* and other *Kazachstania* species) as well as consortia of LAB species with a glucose-repressed maltose metabolism and maltose-positive yeasts [e.g., *Lactiplantibacillus plantarum* (formerly known as *Lactobacillus plantarum*) and *Saccharomyces cerevisiae*], also supporting on mutual relationships (Guerzoni et al., 2007; De Vuyst et al., 2017; Sieuwerts et al., 2018).

Up to now, more than 90 different LAB species and more than 40 different yeast species have been isolated from sourdoughs worldwide (De Vuyst et al., 2017). Yet, a certain backslopped sourdough usually harbors three or less LAB species and one to two yeast species, underlining the competitive ecosystem's mutual relationships and/or matrix-specific adaptations (De Vuyst et al., 2017; Van Kerrebroeck et al., 2017). Alternatively, the microbial composition of sourdoughs depends on the process technology or way of inoculation applied (Gänzle and Ripari, 2016; Van Kerrebroeck et al., 2017). Consequently, a wide variety of traditional sourdoughs exists, some of which have got the annotations of protected designation of origin (PDO; e.g., Italian Altamura bread and Tuscan bread; Ricciardi et al., 2005; Minervini et al., 2012a; Palla et al., 2017) or protected geographical indication (PGI; e.g., Matera bread and *Coppia ferrarese* bread; Vernocchi et al., 2004; Minervini et al., 2012a). Besides tens of studies on Italian sourdough varieties, spontaneous sourdoughs from Belgium, China, France, and Germany have been studied extensively (De Vuyst et al., 2014, 2017; Van Kerrebroeck et al., 2017). Most wheat sourdoughs encountered in these studies harbor at least the LAB species *Lacp. plantarum* and the yeast species *S. cerevisiae*. However, both complex and restricted microbial species diversities have been reported in wheat sourdoughs (Scheirlinck et al., 2008; Minervini et al., 2012a; De Vuyst et al., 2014, 2017; Lhomme et al., 2015a,b). For example, sourdoughs characterized by *Fl. sanfranciscensis* and *K. humilis* solely and sourdoughs containing multiple microbial species, some of which are rarely reported in sourdoughs, such as *Companilactobacillus heilongjiangensis* (formerly known as *Lactobacillus heilongjiangensis*), *Levilactobacillus koreensis* (formerly known as *Lactobacillus koreensis*), and *Lactiplantibacillus xiangfangensis* (formerly known as *Lactobacillus xiangfangensis*), exist (Zhang and He, 2013; Michel et al., 2016; De Vuyst et al., 2017). However, these species are relatively new; therefore, it is possible that they have been misidentified and thus are underrepresented in sourdoughs.

During the last decade, it became clear that also acetic acid bacteria (AAB) are often part of the microbial consortium of spontaneous sourdoughs (Minervini et al., 2012b; Zhang and He, 2013; Lhomme et al., 2015a; Li et al., 2016; Liu et al., 2016; Ripari et al., 2016b; Comasio et al., 2019). However, as they have not been searched for systematically, it is not clear how widespread they are in sourdoughs. Yet, they do not dominate the final sourdoughs, possibly due to their aerobic metabolism. Alternatively, functional starter culture strains of AAB have been tested successfully regarding their exopolysaccharide production in the presence of sucrose (Hermann et al., 2015; Ua-Arak et al., 2016, 2017) or aroma formation potential (Ripari et al., 2016a; Ua-Arak et al., 2017). Finally, alternative yeasts are being exploited to produce innovative sourdoughs (De Vuyst et al., 2016).

The present study aimed to explore the species diversity of sourdoughs from different origins multiphasically, obtained through spontaneous fermentation and maintained through backslopping on a household or bakery scale, to get insight into the communality or uniqueness of the microbial communities involved in sourdough production.

## Materials and Methods

### Collection of Sourdoughs

Seventeen sourdoughs, which were propagated using a backslopping procedure, were collected randomly from private persons as well as from artisan bakeries and a lambic brewery. A specific code was allocated to the different samples (Table 1). All sourdoughs had a dough yield [DY or (dough mass/flour mass) ×100] below 250.

**Table 1:** Characteristics of 17 sourdough samples from backslopped sourdoughs of different origins collected during the present study.

**Type of producer**	**Producer**	**Country**	**Flour used**	**Backslopping time (h)**	**Backslopping water and temperature T**	**Sample code [producer; country (B, Belgium; F, France; UK, United Kingdom; NY, USA); flour used (B, buckwheat; M, multigrain; R, rye; S, spelt; W, wheat; WW, whole wheat); L, lambic beer]**
Household	A	United Kingdom	Rye	na	na	A-UK-R
	B	Belgium	Rye	24–48	Warm water and room T	B-B-R
	C	Belgium	Rye	na	na	C-B-R
Artisan bakery	D	France	Buckwheat	na	na	D-F-B
			Kamut	na	na	D-F-K
			Rye	na	na	D-F-R
			Spelt	na	na	D-F-S
			Wheat	na	na	D-F-W
	E	Belgium	Wheat	24–72	Warm water and room T	E-B-W
			Multigrain	24–72	Warm water and room T	E-B-M
			Rye	24–72	Warm water and room T	E-B-R
			Spelt	24–72	Warm water and room T	E-B-S
	F	USA	Wheat (90%) + rye (10%)	5–8.5–10.5	Water of 3–32–32°C and room T	F-NY-WR
			Whole wheat	9–15	Water of 13–16°C and room T	F-NY-WW
			Rye	10–14	Water of 13–16°C and room T	F-NY-R
Lambic brewery	G	Belgium	Wheat	24	Warm water and room T	G-B-W
			Wheat + lambic beer^*^ (50%)	24	Warm water and room T	G-B-WL

* *Lambic beer is an acidic beer that is the result of a spontaneous fermentation caused by dedicated species of yeasts, lactic acid bacteria, and acetic acid bacteria (De Roos and De Vuyst, 2018, 2019; De Roos et al., 2018b); na, not available*.

### Culture-Dependent Microbiological Analysis

#### Microbial Community Enumeration

To determine the counts of presumptive LAB, AAB, yeasts, and/or cycloheximide-resistant yeasts (only for the sourdoughs G-B-W and G-B-WL) in the sourdough samples, a culture-dependent plating analysis was performed. Therefore, appropriate decimal dilutions of fresh sourdough samples were made and 100 μL of each dilution was plated on (i) modified de Man-Rogosa-Sharpe (mMRS-5) agar medium (Harth et al., 2016), supplemented with 0.1 g of cycloheximide (Sigma-Aldrich, Saint-Louis, Missouri, USA) and 0.005 g of amphotericin B (Sigma-Aldrich); (ii) modified deoxycholate-mannitol-sorbitol (mDMS) agar medium (Papalexandratou et al., 2013), containing 0.1 g of cycloheximide (Sigma-Aldrich) and 0.005 g of amphotericin B (Sigma-Aldrich); (iii) yeast extract-peptone-dextrose (YPD) agar medium, supplemented with 0.1 g of chloramphenicol (Sigma-Aldrich) (Spitaels et al., 2014c); and (iv) YPD agar medium containing 0.1 g of chloramphenicol (Sigma-Aldrich) and 0.05 g of cycloheximide (Sigma-Aldrich) (YPDc; Spitaels et al., 2014c) for sourdough samples of the lambic brewery, respectively. All plates were incubated at 30°C for 72 h (up to 8 days for YPDc). All platings were performed in triplicate. The average counts are expressed as colony forming units (CFU) per g of sourdough.

Up to 32 colonies were randomly picked from the lowest countable dilutions on the mMRS-5, mDMS, YPD, and YPDc agar media. These colonies were grown in 10-mL tubes containing mMRS-5, medium mannitol-yeast extract-peptone medium (MYP; Moens et al., 2014), and YPD media, respectively, at 30°C for 24 h. These cultures (2.0 mL) were stored in cryovials supplemented with glycerol (final concentration of 25%, v/v, Sigma-Aldrich) at −80°C, until further identification.

#### Microbial Identifications

##### Bacterial identifications

Bacterial DNA extraction was performed on cell pellets of all overnight cultures mentioned above, which were obtained through microcentrifugation (14,000× *g*, 5 min), as described previously (Comasio et al., 2019).

To classify and identify the bacteria (GTG)_5_-PCR fingerprinting analysis of genomic DNA was performed, as described previously (Harth et al., 2016). Therefore, the oligonucleotide (GTG)_5_ primer was used (Versalovic et al., 1994; Gevers et al., 2001). PCR assays were performed as described previously (Harth et al., 2016). All fingerprint images were analyzed using Bionumerics v5.10 software (Applied Maths, Sint-Martens-Latem, Belgium). A cluster analysis of the (GTG)_5_-PCR fingerprints was performed using the Pearson correlation coefficient. To calculate a similarity matrix, the intensity of each band of the fingerprints was taken into account. The dendrograms were obtained by means of the unweighted pair group method with arithmetic average (UPGMA) clustering algorithm. Representative isolates (10%) of each cluster were identified by 16S rRNA gene sequencing, after amplification of this gene using the primers pA and pH (Edwards et al., 1989). In the case of AAB isolates, the *dna*K gene was amplified too, using the primers dnaK-01-F and dnaK-02-R (Cleenwerck et al., 2010). The PCR amplicons obtained (*circa* 1,500 and 750 bp for the 16S rRNA gene and the *dna*K gene, respectively) were sequenced in a commercial facility using capillary technology (Macrogen, Amsterdam, The Netherlands; VIB Genetic Service Facility, Antwerp, Belgium). A basic local alignment search tool (BLAST) analysis was performed to evaluate the sequencing results and to determine the closest known relatives (type strains) of the partial gene sequences obtained *via* the National Center for Biotechnology Information (NCBI) non-redundant nucleotide (nt) database (http://www.ncbi.nlm.nih.gov/BLAST/; Altschul et al., 1990). Sequence identities of ≥98% were taken into account. Percentages of identity and accession numbers of hits (GenBank) are reported below.

##### Yeast identifications

Yeast DNA extraction was performed on cell pellets of all overnight yeast cultures mentioned above, which were obtained through microcentrifugation (14,000× g, 5 min), as described previously (Comasio et al., 2019).

To classify and identify the yeasts, M13-PCR fingerprinting analysis of genomic DNA was performed, as described previously (Laureys and De Vuyst, 2014). Therefore, the oligonucleotide primer M13 was used (Vassart et al., 1987). PCR assays were performed as described previously (Harth et al., 2016). M13-PCR fingerprint cluster analysis was performed as described above. At least 10% of representative isolates from each cluster were identified by sequencing of the internal transcribed spacer (ITS) region of the rDNA. Therefore, the primers ITS1 and ITS4 were used (White et al., 1990). The PCR amplicons obtained (variable lengths) were processed as described above. Sequence identities of ≥98% were taken into account. Percentages of identity and accession numbers of hits (GenBank) are reported.

### Culture-Independent Microbial Community Dynamics and Identifications

To determine the prevailing LAB, AAB, and yeast communities present in the different sourdough samples and to compare those results with the culture-dependent data, denaturing gradient gel electrophoresis (DGGE) and high-throughput sequencing (both metagenetics and shotgun metagenomics) were performed.

#### Sample Preparation

Sample preparation was performed as described previously (Harth et al., 2016). For the isolation of total DNA from the sourdough samples, 10 g of sourdough and 90 mL of peptone-physiological solution [0.1%, m/v, bacteriological peptone (Oxoid) and 0.85%, m/v, NaCl (Merck)] were mixed in a stomacher bag (Stomacher 400; Seward, Worthing, West Sussex, UK) for 5 min. These suspensions (50 mL) were centrifuged (1,000 × g for 5 min at 4°C) to remove solid flour particles. The supernatants were collected and a second centrifugation (4,600 × g for 20 min at 4°C) yielded cell pellets that were stored at −20°C.

#### DGGE Analysis

Total DNA extraction was performed as described previously (Laureys and De Vuyst, 2014; Harth et al., 2016). DGGE analysis of PCR amplicons of these total DNA extracts was performed as described previously (Harth et al., 2016). Amplification of the V3 region of the bacterial 16S rRNA gene was done with the universal primers F357-GC and 518R (Muyzer et al., 1993). GC stands for the attached clamp. To amplify fungal DNA from the D1 region of the fungal 26S rRNA gene, the eukaryotic universal primers NL1-GC and LS2 were used (Cocolin et al., 2000). These PCR amplifications were performed as described previously (Comasio et al., 2019).

The PCR amplicons were analyzed by means of a polyacrylamide gel [polyacrylamide, 8% (v/v) in 1 × Tris-acetate-ethylenediaminetetraacetic acid (EDTA) buffer; Bio-Rad, Hercules, California, USA] through DGGE, as described previously (Comasio et al., 2019). The gels were normalized by using ladders of known bacterial and fungal DNA. Therefore, a mixture of PCR products originating from pure cultures of the strains *Lactobacillus amylovorus* DCE 471, *Companilactobacillus crustorum* LMG 23699 (formerly known as *Lactobacillus crustorum*), *Limosilactobacillus fermentum* IMDO 130101 (formerly known as *Lactobacillus fermentum*), *Levilactobacillus namurensis* LMG 23584 (formerly known as *Lactobacillus namurensis*), *Lacp. plantarum* IMDO 130201, *Latilactobacillus sakei* IMDO CG1 (formerly known as *Lactobacillus sakei*), and *Fl. sanfranciscensis* IMDO 150101 (laboratory collection of the research group IMDO) was used to analyze the LAB community profiles. To analyze the fungal community profiles, a ladder was constructed based on PCR products from pure cultures of the strains *S. cerevisiae* DIV/07-125X, *Candida glabrata* DIV/07-076BZ, *Wickerhamomyces anomalus* DIV/07-076BY, and *Kazachstania unispora* DIV/07-125CR (laboratory collection of the research group IMDO). DNA bands of interest were excised from the gels with a sterile blade, resuspended in 30 μL of ultrapure water (MilliQ; Merck Millipore, Burlington, Massachusetts, USA), and incubated at 4°C for 48 h for DNA elution. Five μL of these DNA solutions was used to reamplify the PCR products using the F357-518R primers for the bacteria and the NL1-LS2 primers for the yeasts (both with a M13-tag and no GC clamp; Integrated DNA Technologies, Leuven, Belgium). The M13-tag was used to increase the quality of the sequences of the short PCR products. The amplicons were purified with the Wizard® SV Gel and PCR Clean up system (Promega, Madison, Wisconsin, USA) and sequenced in a commercial facility by means of capillary sequencing technology (Macrogen; VIB Genetic Service Facility). A BLAST analysis was performed to evaluate the sequencing results and to determine the closest known relatives (type strains) of the partial sequences obtained in the NCBI non-redundant nt database (http://www.ncbi.nlm.nih.gov/BLAST/). Sequence identities of ≥98% were taken into account. Percentages of identity and accession numbers of hits (GenBank) are reported below.

#### Metagenetic Sequencing

##### Total DNA extraction, PCR amplification, and DNA sequencing

The same total DNA extracted using the protocol described above was used for metagenetic sequencing. PCR amplifications of group-specific loci of both bacterial and fungal DNA sequences in the total DNA extracted were performed, as described before (De Bruyn et al., 2017; De Roos et al., 2018a). The hypervariable V4 region of the bacterial 16S rRNA gene was amplified using the primers F515 (5′-TCG TCG GCA GCG TCA GAT GTG TAT AAG AGA CAG GTG TGC CAG CMG CCG CGG TAA-3′) and R806 (5′-GTC TCG TGG GCT CGG AGA TGT GTA TAA GAG ACA GGG ACT ACH VGG GTW TCT AAT-3′) with an Illumina platform-specific 5′-tag (underlined) (Caporaso et al., 2011). The fungal ITS1 region was amplified using the primers BITS (5′-TCG TCG GCA GCG TCA GAT GTG TAT AAG AGA CAG ACC TGC GGA RGG ATC A-3′) and B58S3 (5′-GTC TCG TGG GCT CGG AGA TGT GTA TAA GAG ACA GGA GAT CCR TTG YTR AAA GTT-3′) with an Illumina platform-specific 5′-tag (underlined) (Bokulich and Mills, 2013). The PCR amplicons obtained were purified using the Wizard SV Gel and PCR Clean up system (Promega), eluted in 50 μL of nuclease-free water (Promega), and the primer dimers were removed using Agencourt AMPure XP PCR Purification magnetic beads (Beckman Coulter, Brea, California, USA), following the manufacturers' instructions. The amplicon size distribution was checked qualitatively by means of a 2100 Bioanalyzer instrument (Agilent Technologies, Santa Clara, California, USA). Finally, DNA concentrations were quantified using the fluorometric Qubit 2.0 quantitation assay (Thermo Fisher Scientific, Waltham, Massachusetts, USA). Next, the bacterial and fungal DNA template libraries of each sample were combined and sequenced under the same index (De Bruyn et al., 2017). Therefore, bacterial V4 and fungal ITS1 amplicons originating from the same sample were pooled equimolarly in a final volume of 30 μL and barcoded with the same index before being sequenced using an Illumina MiSeq platform (Illumina, San Diego, California, USA) in the interuniversity VUB-ULB sequencing facility BRIGHTcore (Jette, Belgium). All sequences generated were submitted to the European Nucleotide Archive of the European Bioinformatics Institute (ENA/EBI) under accession number PRJEB35796 (experiments ERX3762279-95 for V4 sequences and ERX3762296-312 for ITS1 sequences).

##### Bioinformatic analysis

A trimming of the sequences was performed using Cutadapt software 1.9.6. to remove the adapters (Martin, 2011). Both the bacterial and fungal diversities were processed with DADA2 software v1.6.0, following the pipeline described before (Callahan et al., 2017), yielding amplicon sequence variants (ASVs). The unique bacterial ASVs were aligned against the bacterial 16S rRNA SILVA database (http://www.arb-silva.de; version 128; Quast et al., 2013), whereas the fungal ASVs were aligned against the UNITE_ITS1 database (https://unite.ut.ee; version 6, sh 99; Kõljalg et al., 2005). The resulting sequences of the V4 and ITS1 regions were also aligned using the BLAST tool to determine the closest known relatives (type strains) of the partial sequences obtained in the NCBI non-redundant nt database (http://www.ncbi.nlm.nih.gov/BLAST/). Sequence identities of ≥98% were considered for species classification. When the ASVs resulted in an identical taxonomic assignment, the sequences were merged to determine the relative abundance.

#### Shotgun Metagenomic Sequencing

Shotgun metagenomic sequencing was performed on the four sourdough samples (E-B-W, E-B-M, E-B-R, and E-B-S) from bakery producer E only. High-quality DNA extraction and further processing, as well as taxonomic analysis, were carried out as described below (Laureys and De Vuyst, 2014; Verce et al., 2019).

##### DNA extraction for shotgun metagenomic analysis

For DNA extraction from these four sourdough samples, cell pellets obtained as described above (Section Sample Preparation) were resuspended in 500 μL of lysis buffer (sucrose, 80 g/L; EDTA, 50 mM; Tris-base, 50 mM; lysozyme, 20 mg/mL; pH 8.0), containing 10 μL of mutanolysin (12.5 U/mL), and β-mercaptoethanol (30 mM). These mixtures were incubated at 37°C for 45 min. Afterwards, lyticase (200 U; Sigma-Aldrich) and Zymolyase (15 U; G Biosciences, Saint-Louis, Missouri, USA) were added and the mixtures were incubated at 37°C for another 45 min. Then, 0.2 g of acid-washed 0.2-mm glass beads, 50 μL of SDS solution (20%, m/v), and 50 μL of proteinase K solution (5 mg/mL) were added to the mixtures and the tubes were vortexed for 1 min, followed by an incubation at 56°C for 45 min. One volume of phenol:chloroform:isoamylalcohol (49.5:49.5:1.0) was added to these suspensions, which were then mixed for 1 min and centrifuged at 6,000 × g for 15 min. The aqueous phase was transferred to 15-mL centrifuge tubes, after which one volume of AL buffer (Qiagen, Hilden, Germany) and one volume of absolute ethanol (VWR International, Darmstadt, Germany) were added. The mixtures were repeatedly applied to a DNeasy Blood and Tissue column in aliquots of 600 μL, until the whole mixtures were used, followed by DNA purification with the DNeasy Blood and Tissue Kit (Qiagen), and final elution in 200 μL of elution buffer. An RNase treatment was performed by the addition of 4 μL of RNase (Fermentas, St. Leon-Roth, Germany) to 180 μL of DNA solution and a second purification using the DNeasy Blood and Tissue Kit. The resulting DNA concentrations were measured as described above.

##### Preparation of libraries, shotgun metagenomic sequencing, and data preprocessing

The metagenomic DNA of the four extractions was processed as described previously (Verce et al., 2019). Briefly, they were enzymatically sheared to produce library fragments of the desired length, using an Ion Xpress Plus gDNA Fragment Library Preparation Kit (Thermo Fisher Scientific). First, the shearing time was optimized (5, 8, or 12 min) by following the shearing protocol of the manufacturer. After optimization of the shearing time, three shearing reactions were performed, using an Ion Xpress Plus Fragment Library Kit (Thermo Fisher Scientific), with 100 ng of DNA as input, and the sheared DNA was purified using an Agencourt AMPure XP Kit (Beckman Coulter) according to the manufacturers' instructions. Adapters were ligated to the fragments and the nicks were repaired, followed by another purification of the DNA fragments, using an Agencourt AMPure XP Kit (Beckman Coulter). The three shearing reaction products were pooled during the elution step to ensure a sufficient DNA library concentration. The unamplified DNA library was size-selected using an E-Gel SizeSelect 2.0% (m/v) agarose gel (Thermo Fisher Scientific) to produce library fragments of ~400 bp. The size-selected library was qualified and quantified using a Bioanalyzer 2100 with a High Sensitivity DNA Kit (Agilent Technologies). As such, four 350-bp libraries were obtained for sequencing.

The size-selected libraries were used as template for emulsion PCR onto Ion Sphere Particles (ISPs) using an Ion PGM Template OT2 HiQ View and the Ion OneTouch 2 Instrument (Thermo Fisher Scientific). The template-positive ISPs were measured using the Ion Sphere Quality control kit and were enriched using the Ion PGM Enrichment Beads and an Ion OneTouch ES (Thermo Fisher Scientific). Template-positive ISPs were loaded on an Ion 316 Chip and sequencing was performed using the Ion Hi-Q Sequencing Kit on an Ion PGM (Thermo Fisher Scientific).

The four metagenomic sequence datasets were subjected to quality checks and quality trimming using FastQC v0.10.1 (Andrews, 2012) and PRINSEQ 0.20.2 (Schmieder and Edwards, 2011) with the appropriate settings. All four metagenomic data sets were submitted to the ENA/EBI under accession number PRJEB35796 (experiments ERX3762343-46).

##### Taxonomic analysis of the metagenomic sequence data

The quality-checked metagenomic sequence reads were used to assess the taxonomic composition of the sourdough samples E-B-W, E-B-M, E-B-R, and E-B-S, using BLAST (Altschul et al., 1990), DIAMOND (Buchfink et al., 2015), Kraken (Wood and Salzberg, 2014), and Kaiju (Menzel et al., 2016), as described previously (Verce et al., 2019).

The BLAST algorithm megablast was used to compare the metagenomic reads to sequences in the non-redundant nt database of NCBI (accessed February 2017). DIAMOND was used to compare the metagenomic reads to sequences in the NCBI non-redundant protein (nr) database (accessed September 2017). The outputs were parsed with MEGAN 6.7.11 (Huson et al., 2016), using the following settings: MinScore, 100 (50 for nr); MaxExpected, 0.01; TopPercent, 10.0; MinSupport, 0.01% of all reads; and lowest common ancestor (LCA) percentage, 100.

A database constructed from complete genomes of bacteria, archaea, and fungi, available in GenBank (accessed September 2017) was used for sequence classification with Kraken. A database consisting of protein sequences from the NCBI non-redundant nr database, including microbial eukaryotic sequences, was used for sequence classification with Kaiju.

To construct metagenomic recruitment plots for species-level taxonomic analysis, genera represented by more than 0.1% of all reads in any of the four metagenomes with any of the aforementioned methods were selected, together with the yeast genera detected with culture-dependent methods. Of all species and subspecies of these genera, the genome sequences of the sequenced type strains were obtained from the NCBI RefSeq assembly database (Tatusova et al., 2014). If the genome sequence of the type strain of a species was not available, another representative for that species was chosen, preferably a strain with a complete genome. A BLAST search was performed using blastn, whereby the metagenomic sequence reads were used as query sequences and the genome sequences as database. The minimum identity threshold was set at 60%. Only the top hit for each sequence was retained, using 50 bp as the minimum length. The result was used as a basis for the metagenomic recruitment plotting, using R (R Core Team, 2017), RStudio (RStudio Team, 2015), and the R packages ggplot2 (Wickham, 2009), reshape2 (Wickham, 2017), and scales (Wickham, 2016).

### Metabolite Target Analysis

#### Sample Preparation

To measure the concentrations of residual substrates and metabolites produced in the different sourdough samples, these samples were diluted 5 to 10 times in ultrapure water (MilliQ) and mixed by means of a rotator Stuart SB3 (Bibby Scientific, Stone, Staffordshire, UK) at 25 rpm for 20 min, before being centrifuged (4,600 × g for 20 min) to store the cell-free supernatants at −20°C until further analysis.

#### Determination of Substrate and Metabolite Concentrations

Concentrations of erythritol, fructose, glucose, glycerol, maltose, mannitol, sorbitol, and sucrose were determined by high-performance anion exchange chromatography with pulsed amperometric detection (HPAEC-PAD); those of acetic acid, acetoin, diacetyl, ethanol, and ethyl acetate by gas chromatography with flame ionization detection (GC-FID); and those of L-lactic acid and D-lactic acid by high-performance liquid chromatography with ultraviolet detection (HPLC-UV), as described previously (Comasio et al., 2019).

### Statistical Analysis

Intra-species diversity (alpha-diversity; based on the metagenetic analysis) of both bacteria and yeasts was evaluated by calculating the Simpson (diversity) and Pielou (evenness) indexes. A principal component analysis (PCA) was performed on the quantitative normalized data of the culture-dependent LAB, AAB, and yeast species identifications. A Spearman correlation matrix was calculated based on the microbial species diversity (metagenetic analysis of species with a relative abundance of > 5.0% in at least one of the samples) and metabolites of the sourdough samples. Analysis and visualization were performed using R (R Core Team, 2017), RStudio (RStudio Team, 2015), and the R packages corrplot (Wei and Simko, 2017), factoextra (Kassambara and Mundt, 2020), ggplot2 (Wickham, 2009), and Hmisc (Harrell, 2018).

## Results

### Culture-Dependent Microbial Community Enumerations and Identifications

#### Enumerations

The 17 sourdough samples from different origins contained presumptive LAB (mMRS-5) and yeasts (YPD); all Belgian sourdough samples, except for sourdough sample C-B-R, contained presumptive AAB (mDMS) too. Presumptive LAB represented the most abundant microbial group; their numbers varied between 8.7 log (CFU/g) (sourdough sample E-B-W) and 9.7 log (CFU/g) (D-F-S). The numbers of the presumptive AAB were highest in the samples from the sourdoughs made in the lambic brewery and varied between 6.2 log (CFU/g) (G-B-WL) and 7.9 log (CFU/g) (G-B-W). The numbers of the presumptive yeasts were more variable. Their counts ranged from 4.5 log (CFU/g) (A-UK-R) to 8.1 log (CFU/g) (G-B-W). The sourdoughs made in the lambic brewery also contained presumptive cycloheximide-resistant yeasts (YPDc), namely 7.6 log (CFU/g) (G-B-W) and 5.6 log (CFU/g) (G-B-WL).

The ratios of the LAB to yeast communities varied from 10:1 to 100:1, except for the sourdough samples A-UK-R (10,000:1) and F-NY-WW (1,000:1).

##### LAB identifications

**Figure 1:**
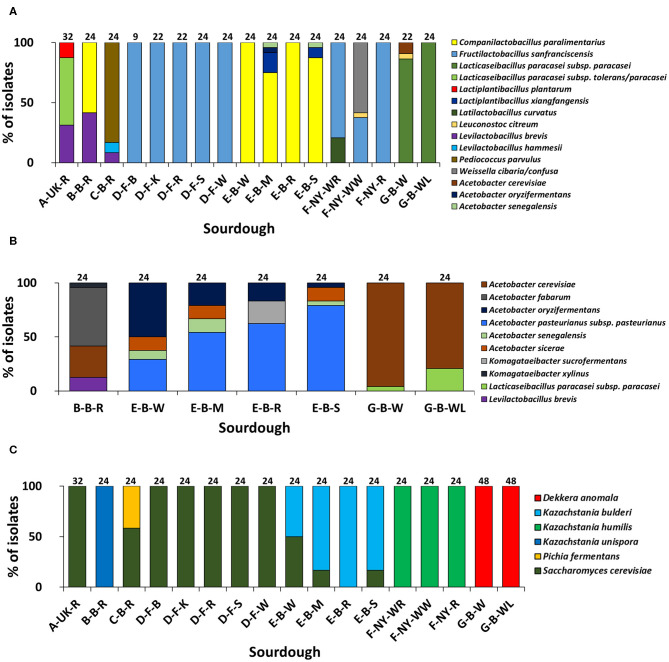
Culture-dependent lactic acid bacterial **(A)**, acetic acid bacterial **(B)**, and yeast **(C)** species diversity of 17 sourdough samples from backslopped sourdoughs of different origins, based on 946 isolates randomly picked from mMRS-5, mDMS, and YPD agar media, respectively. The sample codes are as described in Table 1. The following species were identified: *Levilactobacillus brevis* (99% identity; accession no. LC062897.1); *Latilactobacillus curvatus* (99% identity; accession no. LC063167.1); *Levilactobacillus hammesii* (99% identity; accession no. NR_042243.1); *Lacticaseibacillus paracasei* subsp. *paracasei* (100% identity; accession no. AP012541.1); *Lacticaseibacillus paracasei* subsp. *tolerans/paracasei* (99% identity; accession nos. LC065035.1/AP012541.1); *Companilactobacillus paralimentarius* (99% identity; accession no. LC096230.1); *Lactiplantibacillus plantarum* (100% identity; accession no. NR_113338.1); *Fructilactobacillus sanfranciscensis* (99% identity; accession no. NR_029261.2); *Lactiplantibacillus xiangfangensis* (99% identity; accession no. AB907194.1); *Leuconostoc citreum* (99% identity; accession no. LC096222.1); *Pediococcus parvulus* (99% identity; accession no. NR_113922.1); *Weissella cibaria/confusa* (99% identity; accession nos. LC096236.1/LC063164.1); *Acetobacter fabarum* (99% identity; accession no. HG329536.1); *Acetobacter cerevisiae* (98% identity; accession no. KF537424.1); *Acetobacter pasteurianus* subsp. *pasteurianus* (99% identity; accession no. KF537405.1); *Acetobacter oryzifermentans* (99% identity; accession no. CP011120); *Acetobacter sicerae* (98% identity; accession no. KF537395.1); *Acetobacter senegalensis* (99% identity; accession no. HG424424.1); *Komagataeibacter xylinus* (99% identity; accession no. FN391641.1); *Komagataeibacter sucrofermentans* (99% identity; accession no. FN391639.1); *Dekkera anomala* (99% identity; accession no. KY103306.1); *Kazachstania bulderi* (99% identity; accession no. KY103628.1); *Kazachstania humilis* (98% identity; accession no. KY102142.1); *Kazachstania unispora* (99% identity; accession no. KY103682.1); *Pichia fermentans* (99% identity; accession no. KY104545.1); and *Saccharomyces cerevisiae* (99% identity; accession no. KC881067.1).

Based on (GTG)_5_-PCR fingerprinting and 16S rRNA gene sequencing of colonies picked from the mMRS-5 agar media, different LAB species were identified (Figure 1A). The sourdough sample A-UK-R harbored the species *Lacticaseibacillus paracasei* subsp. *tolerans/paracasei* (formerly known as *Lactobacillus paracasei* subsp. *tolerans/paracasei*; 56.3%), *Lacp. plantarum* (12.5%), and *Levilactobacillus brevis* (formerly known as *Lactobacillus brevis*; 31.2%). The latter species was found in the sourdough sample B-B-R as well (41.7%), together with *Companilactobacillus paralimentarius* (formerly known as *Lactobacillus paralimentarius*; 58.3%). *Fructilactobacillus sanfranciscensis* was the only LAB species found in the five sourdough samples of bakery producer D and was highly prevalent in the three sourdough samples of bakery producer F (79.2% in F-NY-WR; 37.5% in F-NY-WW; and 100.0% in F-NY-R). *Latilactobacillus curvatus* (formerly known as *Lactobacillus curvatus*; 20.8%) or *Leuconostoc citreum* (4.2%) and *Weissella confusa/cibaria* (58.3%) were found in sourdough samples F-NY-WR and F-NY-WW, respectively. Isolates from the two sourdough samples of bakery producer G were identified mainly as *Lacc. paracasei* subsp. *paracasei* (100.0% in G-B-WL; 95.5% in G-B-W), except for one isolate of *Leuc. citreum* (4.5%) that was found in sourdough sample G-B-W. *Companilactobacillus paralimentarius* was found in the four sourdough samples from bakery producer E, whereas *Lacp. xiangfangensis* was isolated only from sourdough samples E-B-M (16.7%) and E-B-S (8.3%).

Sourdough sample C-B-R harbored *Pediococcus parvulus* as most abundant LAB species (83.4%), next to the species *Levl. brevis* (8.3%) and *Levilactobacillus hammesii* (formerly known as *Lactobacillus hammesii*; 8.3%). Few species of LAB were isolated from the mDMS agar media too (Figure 1B), in particular *Levl. brevis* (sourdough samples E-B-W, E-B-M, and E-B-S) and *Lacc. paracasei* subsp. *paracasei* (sourdough samples G-B-W and G-B-WL).

##### AAB identifications

Species of AAB were isolated from the mMRS-5 agar media as well [*Acetobacter cerevisiae* (sourdough sample G-B-W), *Acetobacter oryzifermentans* (E-B-M), and *Acetobacter senegalensis* (E-B-M and E-B-S)] (Figure 1A). In the seven sourdough samples that harbored presumptive AAB, based on mDMS agar plating, different AAB species were identified through (GTG)_5_-PCR fingerprinting and 16S rRNA gene and *dna*K gene sequencing of colonies picked (Figure 1B). *Acetobacter cerevisiae* was the only AAB species isolated from the two sourdough samples of bakery producer G. The same species (29.2%) was found in the sourdough sample B-B-R, together with *Acetobacter fabarum* (54.2%) and *Komagataeibacter xylinus* (4.2%). *Acetobacter pasteurianus* subsp. *pasteurianus* (75.0–83.3%) and *A. oryzifermentans* (4.2–50.0%) were the main AAB species isolated from the four sourdough samples of bakery producer E, whereas *Acetobacter sicerae* (12.5%) and *A. senegalensis* (4.2–12.5%) were present in all sourdough samples of this producer, except for sample E-B-R. In the latter case, *Komagataeibacter sucrofermentans* (20.8%) was isolated.

##### Yeast identifications

The yeast species diversity based on M13-PCR fingerprinting and ITS1/ITS2 sequencing of genomic DNA of colonies picked from the YPD agar media was limited to one to two species per sourdough (Figure 1C). *Saccharomyces cerevisiae* (16.7–50.0%) and *Kazachstania bulderi* (50.0–83.3%) were isolated from three sourdough samples of bakery producer E (E-B-W, E-B-M, and E-B-S); the fourth sourdough sample (E-B-R) contained only *K. bulderi*. *Saccharomyces cerevisiae* was the only yeast species found in the sourdough samples from household producer A and bakery producer D and it co-occurred with *Pichia fermentans* (41.7%) in the sourdough sample C-B-R. *Kazachstania unispora* was found in sourdough sample B-B-R, whereas *K. humilis* was the only yeast species isolated from the three sourdough samples of bakery producer F. *Dekkera anomala* was the only yeast species found in the sourdough samples of lambic brewery producer G, both on the YPD and YPDc agar media.

**Figure 2:**
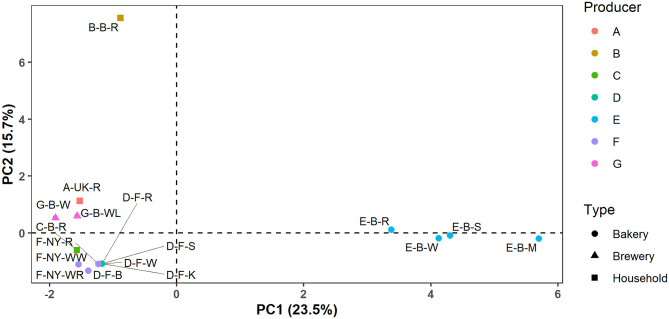
Principal component analysis (PCA) of the normalized culture-dependent lactic acid bacterial, acetic acid bacterial, and yeast species diversity data of 17 sourdough samples from backslopped sourdoughs of different origins. The sample codes are as described in Table 1.

In the case of producers D, E, F, and G, these identification data indicated that the microbial species diversity seemed to be independent of the flour used but was rather similar in sourdoughs coming from the same producer, as further confirmed through a PCA (Figure 2).

### Culture-Independent Microbial Community Identifications

#### rRNA-PCR-DGGE Community Profiles

##### Bacterial community identifications

**Figure 3:**
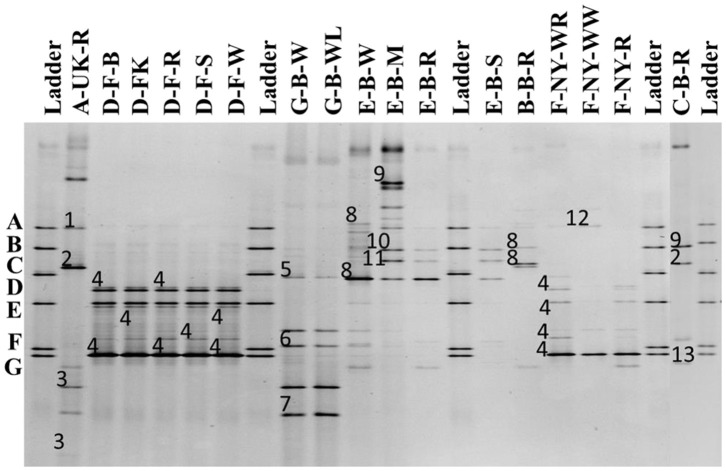
Culture-independent 16S rRNA-PCR-DGGE community profiles of 17 sourdough samples from backslopped sourdoughs of different origins. The sample codes on top of each row are as described in Table 1. The ladder was constructed using pure cultures of *Lactiplantibacillus plantarum* IMDO 130201 **(A)**, *Limosilactobacillus fermentum* IMDO 130101 **(B)**, *Latilactobacillus sakei* IMDO CG1 **(C)**, *Lactobacillus amylovorus* DCE 471 **(D)**, *Companilactobacillus crustorum* LMG 23699 **(E)**, *Levilactobacillus namurensis* LMG 23584 **(F)**, and *Fructilactobacillus sanfranciscensis* IMDO 150101 **(G)**. The following species were identified: 1, *Lacp. plantarum* (100% identity; accession no. KT025937.1); 2, *Levilactobacillus brevis* (99% identity; accession no. LC062897.1); 3, *Lacticaseibacillus rhamnosus/paracasei* subsp. *paracasei/casei* (100% identity; accession nos. LC145553.1/LC096209.1/LC064894.1); 4, *Fl. sanfranciscensis* (100% identity; accession no. NR_029261.2); 5, *Latilactobacillus graminis/curvatus* (100% identity; accession nos. LC097076.1/LC063167.1); 6, *Acetobacter* spp. (98% identity); 7, *Lacc. rhamnosus/paracasei* subsp. *paracasei/casei* (98% identity; accession nos. LC145553.1/LC096209.1/LC064894.1); 8, *Companilactobacillus alimentarius* (99% identity; accession no. LC063166.1); 9, *Levilactobacillus koreensis/yonginensis/hammesii* (100% identity; accession nos. LC145563.1/NR_109452.1/NR_042243.1); 10, *Lacp. plantarum/pentosus/xiangfangensis* (99% identity; accession nos. KT025937.1/KX886789.1/AB907194.1); 11, *Levilactobacillus senmaizukei/parabrevis* (99% identity; accession nos. NR_114251.1/NR_042456.1); 12, *Weissella confusa/cibaria* (100% identity; accession nos. LC063164.1/LC096236.1); and 13, *Pediococcus inopinatus/parvulus/damnosus* (100% identity; accession nos. LC145572.1/LC071841.1/NR_042087.1).

The 16S rRNA-PCR-DGGE profiles of the bacterial communities (Figure 3) present in the sourdough samples analyzed showed similar identifications as those obtained through culture-dependent analysis. This was especially the case for the sourdough sample A-UK-R (*Levl. brevis, Lacp. plantarum*, and *Lacc. paracasei* subsp. *tolerans/paracasei*), the sourdough samples of bakery producer D (*Fl. sanfranciscensis*), and the sourdough samples of bakery producer F (*Fl. sanfranciscensis* in the three sourdough samples analyzed and *W. confusa/cibaria* in sourdough sample F-NY-WW). Next to *Lacc. paracasei* subsp. *paracasei*, 16S rRNA-PCR-DGGE analysis also retrieved the species *Latilactobacillus graminis/curvatus* (formerly known as *Lactobacillus graminis/curvatus*) from the two sourdough samples of lambic brewery producer G. *Companilactobacillus alimentarius* (formerly known as *Lactobacillus alimentarius*) was found in the sourdough samples of household producer B and bakery producer E, which was identified culture-dependently as the closely related *Coml. paralimentarius*. Whereas, sourdough sample E-B-M also contained *Levilactobacillus koreensis/yonginensis/hammesii* (formerly known as *Lactobacillus koreensis/yonginensis/hammesii*), all the sourdough samples from bakery producer E harbored both *Lacp. plantarum/pentosus/xiangfangensis* and *Levilactobacillus senmaizukei/parabrevis* (formerly known as *Lactobacillus senmaizukei*/*parabrevis*). The bacterial species identified in sourdough sample C-B-R were *Levl. brevis, Levl. koreensis/yonginensis/hammesii*, and *Pediococcus inopinatus/parvulus/damnosus*, confirming the LAB species found culture-dependently. AAB were hardly found, except for a species of *Acetobacter* in the sourdough samples of lambic brewery producer G, the identity of which could not be defined.

##### Yeast community identifications

**Figure 4:**
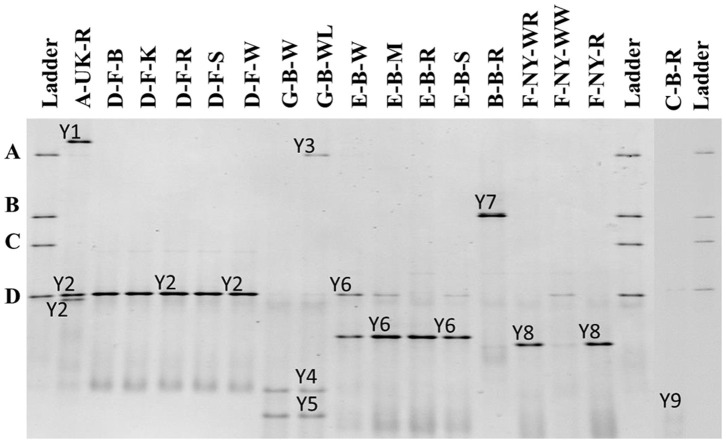
Culture-independent 26S rRNA-PCR-DGGE community profiles of 17 sourdough samples from backslopped sourdoughs of different origins. The sample codes on top of each row are as described in Table 1. The ladder was constructed using pure cultures of *Wickerhamomyces anomalus* DIV/07-076BY **(A)**, *Kazachstania unispora* DIV/07-125CR **(B)**, *Candida glabrata* DIV/07-076BZ **(C)**, and *Saccharomyces cerevisiae* DIV/07-125X **(D)**. The following yeast species were identified: Y1, *Pichia myanmarensis* (98% identity; accession no. KY108896.1); Y2, *Saccharomyces cariocanus/paradoxus* (100% identity; accession nos. KY109235.1/BR000309.1); Y3, *Pi. myanmarensis* (99% identity; accession no. KY108896.1); Y4, *Dekkera bruxellensis* (98% identity; accession no. AY969049.1); Y5, *Dekkera anomala* (99% identity; accession no. AY969114.1); Y6, *Kazachstania exigua/pseudohumilis* (100% identity; accession nos. NG_055049.1/KY106702.1); Y7, *Kazachstania solicola* (99% identity; accession no. KY107950.1); Y8, *Kazachstania humilis* (100% identity; accession no. KY106507.1); and Y9, *Pichia fermentans* (99% identity; accession no. KY108804.1).

The 26S rRNA-PCR-DGGE profiles of the yeast communities present in the sourdough samples analyzed confirmed the low species diversity that was shown culture-dependently (Figure 4). *Pichia myanmarensis*, a yeast species closely related to *Wickerhamomyces anomalus*, was present in the sourdough samples A-UK-R and G-B-WL. *Saccharomyces cariocanus/paradoxus* was also found in the former sourdough sample and was the only yeast species found in the sourdough samples from bakery producer D. The brewery sourdough samples G-B-W and G-B-WL harbored the species *D. anomala* (also found culture-dependently) and *Dekkera bruxellensis*. *Kazachstania solicola, Kazachstania exigua/pseudohumilis*, and *K. humilis* were present in sourdough samples from household producer B and bakery producers E and F, respectively, the latter species being found culture-dependently as well. From sourdough sample C-B-R, *Pi. fermentans* could be detected through 16S rRNA-PCR-DGGE analysis.

As for the culture-dependent analysis, the microbial species diversity seemed to be independent of the flour used in the case of the producers D, E, F, and G, but was rather similar in sourdough samples coming from the same producer.

#### Metagenetics

##### Bacterial species diversity

Amplicon sequencing applied on total DNA extracted from all sourdough samples analyzed generally resulted in a greater LAB and AAB species diversity (Tables 2, 3) compared to culture-dependent plating/identification and rRNA-PCR-DGGE community profiling (Table 4). The highest intra-species diversity, based on the Simpson and Pielou indexes, was found in the samples of sourdoughs from household producer A, bakery producer C, and lambic brewery producer G (Table 2). For instance, concerning LAB, ASVs belonging to *Latilactobacillus graminis/fuchuensis* (formerly known as *Lactobacillus graminis*/*fuchuensis*), the *Companilactobacillus* genus (formerly the *Lb. alimentarius* group), and *Fl. sanfranciscensis* were found in the sourdough sample A-UK-R, next to those found culture-dependently and through 16S rRNA-PCR-DGGE analysis. The latter species was also found in the sourdough samples of bakery producer F and as the sole LAB species in those of bakery producer D, as shown culture-dependently and through 16S rRNA-PCR-DGGE analysis. It represented a minority of 7.0% in the sourdough sample C-B-R and <1.0% in the sourdough samples B-B-R, E-B-W, E-B-M, E-B-S, and G-B-W. In the sourdough samples of lambic brewery producer G, mainly *Lacc. paracasei/casei* (as shown culture-dependently and through 16S rRNA-PCR-DGGE analysis), as well as *Latl. sakei/curvatus*, were found. In the rye sourdough sample B-B-R, mainly the *Companilactobacillus* genus and *Levl. brevis* were present, as shown culture-dependently. The *Companilactobacillus* genus was also present in all sourdough samples from bakery producer E, as shown culture-dependently and through 16S rRNA-PCR-DGGE analysis, followed by *Levl. parabrevis/hammesii* (low relative abundance in sourdough sample E-B-W). Reads belonging to the *Lentilactobacillus* genus (formerly the *Lactobacillus buchneri* group) were present in the sourdough samples from bakery producer E too (most in sourdough sample E-B-S). Sourdough sample C-B-R mainly harbored *Levl. parabrevis/hammesii*, followed by *Levl. brevis* and *P. parvulus*, as shown culture-dependently and through 16S rRNA-PCR-DGGE analysis.

**Table 2:** Intra-species diversity of 17 sourdough samples from backslopped sourdoughs of different origins, based on the relative abundances of bacterial and yeast species obtained by metagenetic analysis.

**Sourdough sample**	**Bacteria**		**Yeasts**	
	**Simpson (1-D)**	**Pielou (Je)**	**Simpson (1-D)**	**Pielou (Je)**
A-UK-R	0.60	0.62	0.26	0.39
B-B-R	0.44	0.40	0.01	0.03
C-B-R	0.64	0.54	0.26	0.25
D-F-B	0.00	0.01	0.07	0.08
D-F-K	0.00	0.00	0.01	0.03
D-F-R	0.00	0.00	0.01	0.02
D-F-S	0.00	0.00	0.01	0.03
D-F-W	0.00	0.00	0.01	0.02
E-B-W	0.29	0.32	0.01	0.06
E-B-M	0.65	0.59	0.01	0.03
E-B-R	0.49	0.51	0.01	0.02
E-B-S	0.61	0.55	0.12	0.12
F-NY-WR	0.08	0.14	0.00	0.01
F-NY-WW	0.19	0.34	0.79	0.61
F-NY-R	0.00	0.01	0.00	0.00
G-B-W	0.78	0.70	0.50	0.43
G-B-WL	0.69	0.62	0.51	0.40

*The sample codes are as described in Table 1. The Simpson (1-D) and Pielou (Je) indexes were calculated to measure the sourdough alpha diversity and evenness, respectively*.

**Table 3:** Relative abundance (%) of bacterial amplicon sequence variants (ASVs) obtained through metagenetic analysis of 17 sourdough samples from backslopped sourdoughs of different origins.

**Species/genus identifications**	**Sourdough sample**															
	**A-UK-R**	**B-B-R**	**C-B-R**	**D-F-B**	**D-F-K**	**D-F-R**	**D-F-S**	**D-F-W**	**E-B-W**	**E-B-M**	**E-B-R**	**E-B-S**	**F-NY-WR**	**F-NY-WW**	**F-NY-R**	**G-B-W**	**G-B-WL**
*Companilactobacillus*	0.1	70.8	0.8	0.0	0.0	0.0	0.0	0.0	83.9	28.3	68.6	45.4	0.0	0.0	0.0	0.0	0.0
*Fl. sanfranciscensis*	1.4	0.3	7.0	99.9	100.0	100.0	100.0	100.0	0.2	0.4	0.0	0.3	96.2	89.7	99.9	0.1	0.0
*Ff. rossiae*	0.0	0.0	0.8	0.0	0.0	0.0	0.0	0.0	0.0	0.0	0.0	0.0	0.0	0.0	0.0	0.0	0.0
*Lacc. casei/paracasei*	53.9	0.0	0.0	0.0	0.0	0.0	0.0	0.0	0.0	0.0	0.0	0.2	0.0	0.0	0.0	31.9	46.2
*Lactiplantibacillus*	7.7	2.0	0.3	0.0	0.0	0.0	0.0	0.0	0.0	0.0	0.0	0.0	0.0	0.0	0.0	0.0	0.0
*Lacp. xiangfangensis/modestisalitolerans*	0.0	0.0	0.0	0.0	0.0	0.0	0.0	0.0	0.9	11.9	1.7	2.4	0.0	0.0	0.0	0.0	0.0
*Latilactobacillus*	0.0	0.0	0.0	0.0	0.0	0.0	0.0	0.0	0.0	0.0	0.0	0.0	0.7	0.0	0.0	0.0	0.0
*Latl. curvatus/sakei*	0.0	0.0	0.9	0.0	0.0	0.0	0.0	0.0	0.0	0.0	0.0	0.0	2.7	0.0	0.0	12.6	11.2
*Latl. graminis/fuchuensis*	5.1	0.0	0.0	0.0	0.0	0.0	0.0	0.0	0.0	0.0	0.0	0.0	0.0	0.0	0.0	0.0	0.0
*Lenl. buchneri*	0.0	0.0	0.0	0.0	0.0	0.0	0.0	0.0	0.1	1.7	2.5	6.5	0.0	0.0	0.0	0.0	0.0
*Leuconostoc*	0.0	0.0	0.1	0.0	0.0	0.0	0.0	0.0	0.0	0.0	0.0	0.0	0.0	1.5	0.0	9.1	0.7
*Levl. brevis*	31.8	23.5	22.7	0.0	0.0	0.0	0.0	0.0	0.0	0.0	0.0	0.0	0.0	0.0	0.0	0.0	0.0
*Levl. hammesii/parabrevis*	0.0	0.0	54.3	0.0	0.0	0.0	0.0	0.0	3.1	49.9	17.3	41.7	0.0	0.0	0.0	0.0	0.0
*Levl. paucivorans*	0.0	0.6	0.0	0.0	0.0	0.0	0.0	0.0	0.0	0.0	0.0	0.0	0.0	0.0	0.0	0.0	0.0
*Loil. coryniformis*	0.0	0.0	1.6	0.0	0.0	0.0	0.0	0.0	0.0	0.0	0.0	0.0	0.0	0.0	0.0	0.0	0.0
*P. parvulus*	0.0	0.0	11.0	0.0	0.0	0.0	0.0	0.0	0.0	0.0	0.0	0.0	0.0	0.0	0.0	0.0	0.0
*Weissella confusa* group	0.0	0.0	0.0	0.0	0.0	0.0	0.0	0.0	0.0	0.0	0.0	0.0	0.4	8.8	0.0	0.0	0.0
*A. fabarum*	0.0	0.4	0.0	0.0	0.0	0.0	0.0	0.0	0.0	0.0	0.0	0.0	0.0	0.0	0.0	0.0	0.0
*A. lambici/okinawensis/indonesiensis*	0.0	0.0	0.0	0.0	0.0	0.0	0.0	0.0	0.0	0.0	0.0	0.0	0.0	0.0	0.0	15.6	18.5
*A. malorum/cerevisiae*	0.0	0.3	0.0	0.0	0.0	0.0	0.0	0.0	3.9	1.1	0.0	0.5	0.0	0.0	0.0	27.0	22.1
*A. orleanensis*	0.0	0.0	0.0	0.0	0.0	0.0	0.0	0.0	0.0	0.0	0.0	0.0	0.0	0.0	0.0	0.0	0.7
*A. pasteurianus/ghanensis/oryzifermentans*	0.0	0.0	0.0	0.0	0.0	0.0	0.0	0.0	7.4	6.0	9.1	3.0	0.0	0.0	0.0	0.0	0.0
*Asaia astilbis*	0.0	0.0	0.0	0.0	0.0	0.0	0.0	0.0	0.0	0.0	0.0	0.0	0.0	0.0	0.0	0.0	0.3
*G. frauterii/japonicus*	0.0	0.0	0.0	0.0	0.0	0.0	0.0	0.0	0.0	0.0	0.0	0.0	0.0	0.0	0.0	1.6	0.0
*Komagataeibacter*	0.0	2.1	0.0	0.0	0.0	0.0	0.0	0.0	0.5	0.6	0.5	0.0	0.0	0.0	0.0	0.0	0.0
*Bacillus*	0.0	0.0	0.0	0.0	0.0	0.0	0.0	0.0	0.0	0.0	0.0	0.0	0.0	0.0	0.0	0.3	0.0
*Clostridium* sensu stricto 1	0.0	0.0	0.0	0.0	0.0	0.0	0.0	0.0	0.0	0.0	0.0	0.0	0.0	0.0	0.0	1.1	0.1
*Pantoea*	0.0	0.0	0.4	0.1	0.0	0.0	0.0	0.0	0.0	0.0	0.3	0.0	0.0	0.0	0.0	0.4	0.0
*Pseudomonas*	0.0	0.0	0.0	0.0	0.0	0.0	0.0	0.0	0.0	0.0	0.0	0.0	0.0	0.0	0.0	0.35	0.0
Others	0.0	0.0	0.1	0.0	0.0	0.0	0.0	0.0	0.0	0.1	0.0	0.0	0.0	0.0	0.1	0.0	0.2

*The sample codes are as described in Table 1. ASVs with an occurrence under 0.1 % were grouped as “Others.” The following abbreviations are used: Fl., Fructilactobacillus; Ff., Furfurilactobacillus; Lacc., Lacticaseibacillus; Lacp., Lactiplantibacillus; Latl., Latilactobacillus; Lenl., Lentilactobacillus; Levl., Levilactobacillus; Loil., Loigolactobacillus; P., Pediococcus; A., Acetobacter; G., Gluconobacter. The intensity of the blue color is based on the percentage of reads assigned to a specific species/genus*.

**Table 4:** Comparison of the culture-dependent and culture-independent identifications of lactic acid bacterial and acetic acid bacterial genera in 17 sourdough samples from backslopped sourdoughs of different origins, indicated as presence (+) or absence (–) through colony identification, rRNA-PCR-DGGE, metagenetics, and metagenomics (only producer E), respectively.

**Sourdough sample**	**Genus identifications**																	
	***Companilactobacillus***	***Fructilactobacillus***	***Furfurilactobacillus***	***Lacticaseibacillus***	***Lactiplantibacillus***	***Lactobacillus***	***Latilactobacillus***	***Lentilactobacillus***	***Leuconostoc***	***Levilactobacillus***	***Limosilactobacillus***	***Loigolactobacillus***	***Pediococcus***	***Secundilactobacillus***	***Weissella***	***Acetobacter***	***Asaia***	***Komagataeibacter***	***Gluconobacter***
A-UK-R	–/–/+	–/–/+	–/–/–	+/+/+	+/+/+	–/–/–	–/–/+	–/–/–	–/–/–	+/+/+	–/–/–	–/–/–	–/–/–	–/–/–	–/–/–	–/–/–	–/–/–	–/–/–	–/–/–
B-B-R	+/+/+	–/–/+	–/–/–	–/–/–	–/–/+	–/–/–	–/–/–	–/–/–	–/–/–	+/–/+	–/–/–	–/–/–	–/–/–	–/–/–	–/–/–	+/–/+	–/–/–	+/–/+	–/–/–
C-B-R	–/–/+	–/–/+	–/–/+	–/–/–	–/–/+	–/–/–	–/–/+	–/–/–	–/–/+	+/+/+	–/–/–	–/–/+	+/+/+	–/–/–	–/–/–	–/–/–	–/–/–	–/–/–	–/–/–
D-F-B	–/–/–	+/+/+	–/–/–	–/–/–	–/–/–	–/–/–	–/–/–	–/–/–	–/–/–	–/–/–	–/–/–	–/–/–	–/–/–	–/–/–	–/–/–	–/–/–	–/–/–	–/–/–	–/–/–
D-F-K	–/–/–	+/+/+	–/–/–	–/–/–	–/–/–	–/–/–	–/–/–	–/–/–	–/–/–	–/–/–	–/–/–	–/–/–	–/–/–	–/–/–	–/–/–	–/–/–	–/–/–	–/–/–	–/–/–
D-F-R	–/–/–	+/+/+	–/–/–	–/–/–	–/–/–	–/–/–	–/–/–	–/–/–	–/–/–	–/–/–	–/–/–	–/–/–	–/–/–	–/–/–	–/–/–	–/–/–	–/–/–	–/–/–	–/–/–
D-F-S	–/–/–	+/+/+	–/–/–	–/–/–	–/–/–	–/–/–	–/–/–	–/–/–	–/–/–	–/–/–	–/–/–	–/–/–	–/–/–	–/–/–	–/–/–	–/–/–	–/–/–	–/–/–	–/–/–
D-F-W	–/–/–	+/+/+	–/–/–	–/–/–	–/–/–	–/–/–	–/–/–	–/–/–	–/–/–	–/–/–	–/–/–	–/–/–	–/–/–	–/–/–	–/–/–	–/–/–	–/–/–	–/–/–	–/–/–
E-B-W	+/+/+/+	–/–/+/–	–/–/–/–	–/–/–/+	–/+/+/+	–/–/–/–	–/–/–/–	–/–/+/+	–/–/–/–	–/+/+/+	–/–/–/+	–/–/–/–	–/–/–/–	–/–/–/–	–/–/–/–	+/–/+/+	–/–/–/–	–/–/+/+	–/–/–/–
E-B-M	+/+/+/+	–/–/+/–	–/–/–/+	–/–/–/+	+/+/+/+	–/–/–/+	–/–/–/–	–/–/+/+	–/–/–/–	–/+/+/+	–/–/–/+	–/–/–/–	–/–/–/–	–/–/–/–	–/–/–/–	+/–/+/+	–/–/–/–	–/–/+/+	–/–/–/–
E-B-R	+/+/+/+	–/–/–/–	–/–/–/–	–/–/–/–	–/+/+/+	–/–/–/–	–/–/–/–	–/–/+/+	–/–/–/–	–/+/+/+	–/–/–/–	–/–/–/–	–/–/–/–	–/–/–/–	–/–/–/–	+/–/+/+	–/–/–/–	+/–/+/+	–/–/–/–
E-B-S	+/+/+/+	–/–/+/–	–/–/–/+	–/–/+/+	+/+/+/+	–/–/–/–	–/–/–/–	–/–/+/+	–/–/–/–	–/+/+/+	–/–/–/–	–/–/–/–	–/–/–/–	–/–/–/+	–/–/–/–	+/–/+/+	–/–/–/–	–/–/–/+	–/–/–/–
F-NY-WR	–/–/–	+/+/+	–/–/–	–/–/–	–/–/–	–/–/–	+/–/+	–/–/–	–/–/–	–/–/–	–/–/–	–/–/–	–/–/–	–/–/–	–/–/+	–/–/–	–/–/–	–/–/–	–/–/–
F-NY-WW	–/–/–	+/+/+	–/–/–	–/–/–	–/–/–	–/–/–	–/–/–	–/–/–	+/–/+	–/–/–	–/–/–	–/–/–	–/–/–	–/–/–	+/+/+	–/–/–	–/–/–	–/–/–	–/–/–
F-NY-R	–/–/–	+/+/+	–/–/–	–/–/–	–/–/–	–/–/–	–/–/–	–/–/–	–/–/–	–/–/–	–/–/–	–/–/–	–/–/–	–/–/–	–/–/–	–/–/–	–/–/–	–/–/–	–/–/–
G-B-W	–/–/–	–/–/+	–/–/–	+/+/+	–/–/–	–/–/–	–/+/+	–/–/–	+/–/+	–/–/–	–/–/–	–/–/–	–/–/–	–/–/–	–/–/–	+/+/+	–/–/–	–/–/–	–/–/+
G-B-WL	–/–/–	–/–/–	–/–/–	+/+/+	–/–/–	–/–/–	–/+/+	–/–/–	–/–/+	–/–/–	–/–/–	–/–/–	–/–/–	–/–/–	–/–/–	+/+/+	–/–/+	–/–/–	–/–/–

*The sample codes are as described in Table 1. When a genus was identified in a sample by at least one of the different methods, a green color was used*.

Different AAB species were found in the Belgian sourdough samples, except for the C-B-R sourdough sample. The brewery sourdoughs harbored *Acetobacter malorum/cerevisiae* and *Acetobacter lambici/okinawensis/indonesiensis*. The four sourdough samples of bakery producer E contained mainly *Acetobacter pasteurianus/ghanensis/oryzifermentans*, as shown culture-dependently. Also *Komagataeibacter* was present in some sourdough samples, but with low and decreasing relative abundances in sourdough samples B-B-R, E-B-M, E-B-W, and E-B-R. Low relative abundances of ASVs belonging to the genera *Asaia, Bacillus, Clostridium, Gluconobacter, Pantoea*, and *Pseudomonas* were found as well, especially in the sourdough samples G-B-W and G-B-WL.

##### Fungal species diversity

In contrast with a great bacterial species diversity, amplicon sequencing of total DNA from the sourdough samples revealed a limited yeast species diversity, except for the sourdough sample F-NY-WW (highest intra-species alpha-diversity based on the Simpson and Pielou indexes), which contained different yeast species (in particular *K. humilis*, which was also present in the other sourdough samples of bakery producer F, as shown culture-dependently and through 26S rRNA-PCR-DGGE analysis) as well as filamentous fungi (Tables 2, 5, 6). Filamentous fungi were also present in some other sourdough samples (B-B-R, C-B-R, D-F-B, E-B-W, and E-B-R). In sourdough sample A-UK-R, a high relative abundance of *W. anomalus*, next to *S. cerevisiae* (shown culture-dependently), was found. The producer D sourdoughs mainly contained *S. cerevisiae*, as shown culture-dependently. ASVs belonging to *D. anomala* (shown culture-dependently and through 26S rRNA-PCR-DGGE analysis) and *Pichia membranifaciens* were mainly present in the sourdough samples of lambic brewery producer G. *Kazachstania unispora* was unique for sourdough sample B-B-R, as shown culture-dependently. The sourdough samples from bakery producer E contained mainly *K. bulderi* and *S. cerevisiae* (lower relative abundance), as shown culture-dependently for the respective sourdough samples. Sourdough sample C-B-R mainly harbored *Pi. fermentans*, confirming the culture-dependent and 26S rRNA-PCR-DGGE data.

**Table 5:** Relative abundance (%) of fungal amplicon sequence variants (ASVs) obtained through metagenetic analysis of 17 sourdough samples from backslopped sourdoughs of different origins.

**Species/genus identifications**	**Sourdough sample**																
	**A-UK-R**	**B-B-R**	**C-B-R**	**D-F-B**	**D-F-K**	**D-F-R**	**D-F-S**	**D-F-W**	**E-B-W**	**E-B-M**	**E-B-R**	**E-B-S**	**F-NY-WR**	**F-NY-WW**	**F-NY-R**	**G-B-W**	**G-B-WL**
*Candida quercitrusa*	0.0	0.0	0.0	0.0	0.0	0.0	0.0	0.0	0.0	0.0	0.0	0.0	0.0	2.4	0.0	0.0	0.0
*Dekkera anomala*	0.0	0.0	0.0	0.0	0.0	0.0	0.0	0.0	0.0	0.0	0.0	0.0	0.0	0.0	0.0	48.4	42.4
*Kazachstania bulderi*	0.0	0.0	0.0	0.0	0.0	0.0	0.0	0.0	93.9	99.3	99.3	99.5	0.0	0.0	0.0	0.0	0.0
*Kazachstania humilis*	0.0	0.0	0.0	0.0	0.0	0.0	0.0	0.0	0.0	0.0	0.0	0.0	99.8	41.8	99.9	0.0	0.0
*Kazachstania unispora*	0.0	99.2	0.0	0.0	0.0	0.0	0.0	0.0	0.0	0.0	0.0	0.0	0.0	0.0	0.0	0.0	0.0
*Pichia fermentans*	0.0	0.0	88.6	0.0	0.0	0.0	0.0	0.0	0.0	0.0	0.0	0.0	0.0	0.0	0.0	0.0	0.0
*Pichia kluyveri*	0.0	0.0	0.0	0.0	0.5	0.0	0.5	0.0	0.0	0.0	0.0	0.0	0.0	0.0	0.0	0.0	0.0
*Pichia membranifaciens*	0.0	0.0	0.0	0.0	0.0	0.0	0.0	0.0	0.0	0.0	0.0	0.0	0.0	0.0	0.0	51.6	55.5
*Saccharomyces cerevisiae*	15.3	0.0	0.0	96.2	99.4	99.4	99.5	99.7	4.3	0.7	0.0	0.3	0.0	9.5	0.0	0.0	0.0
*Sporobolomyces roseus*	0.0	0.0	0.0	0.0	0.0	0.0	0.0	0.0	0.0	0.0	0.0	0.0	0.0	5.3	0.0	0.0	0.0
*Vishniacozyma*	0.0	0.0	0.0	0.0	0.0	0.0	0.0	0.0	0.0	0.0	0.0	0.0	0.0	1.0	0.0	0.0	0.0
*Vishniacozyma victoriae*	0.0	0.0	0.0	0.5	0.0	0.0	0.0	0.0	0.0	0.0	0.0	0.0	0.0	8.8	0.0	0.0	0.0
*Wickerhamomyces anomalus*	84.7	0.0	0.0	0.0	0.0	0.0	0.0	0.0	0.4	0.0	0.0	0.0	0.0	0.0	0.0	0.0	2.0
Filamentous fungi	0.0	0.4	10.2	1.7	0.0	0.0	0.0	0.0	0.9	0.0	0.4	0.0	0.0	26.6	0.0	0.0	0.0
Others	0.0	0.4	1.2	1.6	0.1	0.6	0.0	0.3	0.5	0.0	0.3	0.2	0.2	4.6	0.1	0.0	0.1

*The sample codes are as described in Table 1. ASVs with an occurrence below 0.25% (0.5% for F-NY-WW) were grouped as “Others”. ASVs of non-yeast fungal communities were grouped together. The intensity of the blue color is based on the percentage of reads assigned to a specific species/genus*.

**Table 6:** Comparison of the culture-dependent and culture-independent identifications of yeast genera in 17 sourdough samples from backslopped sourdoughs of different origins, indicated as presence (+) or absence (–) through colony identification, rRNA-PCR-DGGE, metagenetics, and metagenomics (only producer E).

**Sourdough sample**	**Genus identifications**							
	***Candida***	***Dekkera***	***Kazachstania***	***Pichia***	***Saccharomyces***	***Sporobolomyces***	***Vishniacozyma***	***Wickerhamomyces***
A-UK-R	–/–/–	–/–/–	–/–/–	–/+/–	+/+/+	–/–/–	–/–/–	–/–/–
B-B-R	–/–/–	–/–/–	+/+/+	–/–/–	–/–/–	–/–/–	–/–/–	–/–/–
C-B-R	–/–/–	–/–/–	–/–/–	+/+/+	+/–/–	–/–/–	–/–/–	–/–/–
D-F-B	–/–/–	–/–/–	–/–/–	–/–/–	+/+/+	–/–/–	–/–/+	–/–/–
D-F-K	–/–/–	–/–/–	–/–/–	–/–/+	+/+/+	–/–/–	–/–/–	–/–/–
D-F-R	–/–/–	–/–/–	–/–/–	–/–/–	+/+/+	–/–/–	–/–/–	–/–/–
D-F-S	–/–/–	–/–/–	–/–/–	–/–/+	+/+/+	–/–/–	–/–/–	–/–/–
D-F-W	–/–/–	–/–/–	–/–/–	–/–/–	+/+/+	–/–/–	–/–/–	–/–/–
E-B-W	–/–/–/–	–/–/–/–	+/+/+/+	–/–/–/–	+/–/+/+	–/–/–/–	–/–/–/–	–/–/+/–
E-B-M	–/–/–/–	–/–/–/–	+/+/+/+	–/–/–/–	+/–/+/+	–/–/–/–	–/–/–/–	–/–/–/–
E-B-R	–/–/–/–	–/–/–/–	+/+/+/+	–/–/–/–	–/–/–/–	–/–/–/–	–/–/–/–	–/–/–/–
E-B-S	–/–/–/–	–/–/–/–	+/+/+/+	–/–/–/–	+/–/+/+	–/–/–/–	–/–/–/–	–/–/–/–
F-NY-WR	–/–/–	–/–/–	+/+/+	–/–/–	–/–/–	–/–/–	–/–/–	–/–/–
F-NY-WW	–/–/+	–/–/–	+/+/+	–/–/–	–/–/+	–/–/+	–/–/+	–/–/–
F-NY-R	–/–/–	–/–/–	+/+/+	–/–/–	–/–/–	–/–/–	–/–/–	–/–/–
G-B-W	–/–/–	+/+/+	–/–/–	–/–/–	–/–/–	–/–/–	–/–/–	–/–/–
G-B-WL	–/–/–	+/+/+	–/–/–	–/+/+	–/–/–	–/–/–	–/–/–	–/–/+

*The sample codes are as described in Table 1. When a genus was identified in a sample by at least one of the different methods, a green color was used*.

**Figure 5:**
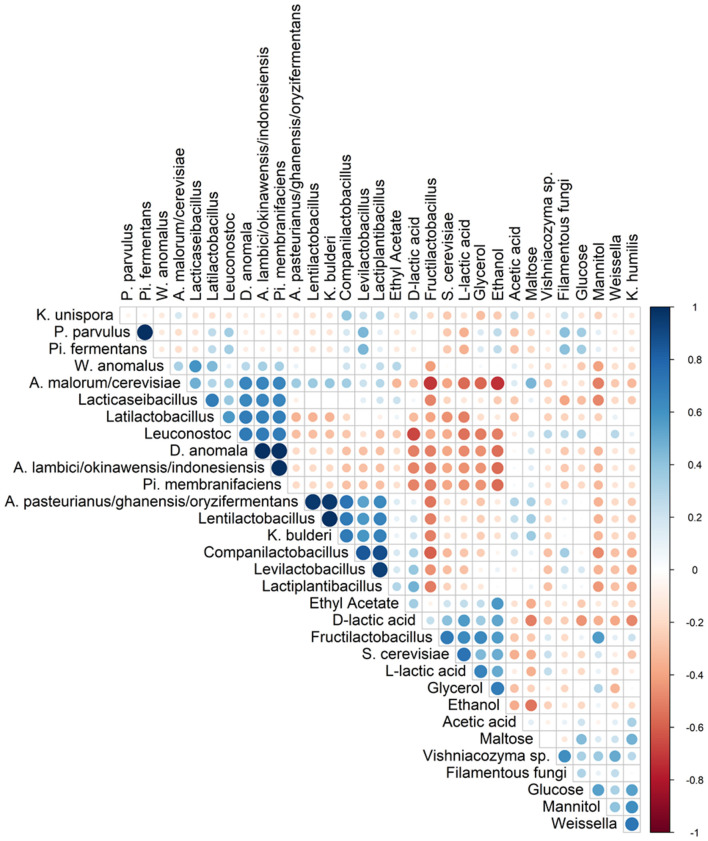
Spearman correlation analysis between the different species (based on metagenetic analysis) and/or dedicated substrates consumed, and metabolites produced in 17 sourdough samples from backslopped sourdoughs of different origins. Positive and negative correlations are represented in blue and red, respectively. The size of the circle represents the numerical value of the correlation coefficient.

Spearman correlation analysis showed that *Fl. sanfranciscensis* negatively correlated with other LAB and AAB species but did positively correlate with *S. cerevisiae* and *K. humilis* (Figure 5).

#### Shotgun Metagenomic Sequencing

The sequencing of the four metagenomic libraries derived from the sourdough samples of bakery producer E resulted in four datasets with a combined size of 4.28 Gbp of metagenomic sequence data after quality checks and trimming. The lower and upper limits of the read lengths were 25 and 372 bp, respectively; the median read length was 283 bp.

Taxonomic analysis of the four metagenomic sequence datasets assigned the reads to the family *Lactobacillaceae* (up to *circa* 70% of the total reads using Kraken and Kaiju), and the genus *Acetobacter* (up to *circa* 15% using Kraken and Kaiju) and *Komagataeibacter* (*circa* 1–2% with all tools applied) as main bacterial taxa. Less than 1% of the total reads were assigned to yeast genera using these taxonomy profiling tools. A low number of reads (<3%) were assigned to fruit flies (*Drosophila*) and nematodes (*Trichinella*) using BLAST and DIAMOND, respectively. Also, depending on the flour used, less than 10 or 2% of the reads were assigned to cereals (*Secale* and *Triticum)* using BLAST and DIAMOND, respectively.

**Figure 6:**
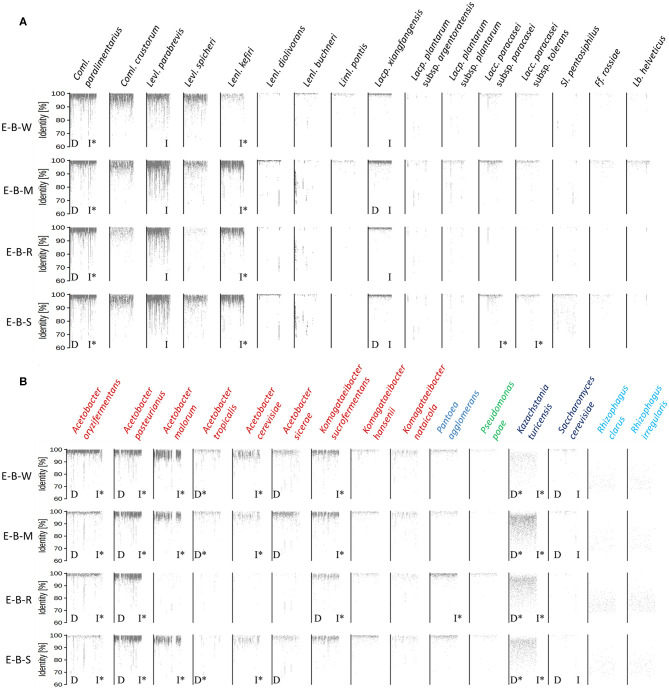
Recruitment plots of metagenomic reads of lactic acid bacteria **(A)** and Gram-negative bacteria and yeast **(B)** species found in the sourdough samples E-B-W, E-B-M, E-B-R, and E-B-S from artisan bakery producer E. Each dot on the recruitment plots represents a bin of reads. The intensity of the color of the dot represents the normalized number of reads in a bin within a genome. Species found culture-dependently (D) and culture-independently through rRNA-PCR-DGGE community profiling or metagenetic analysis (I) are shown. The asterisk (*) is used when species closely related were found with the methods mentioned previously.

The metagenomic recruitment plots of the reads of the four datasets showed a high inter- and intra-species diversity (Figure 6 and Supplementary Table 1). However, sourdough sample E-B-R carried fewer microbial species compared to the three other samples of bakery producer E. For the four bakery E sourdough samples, many reads were assigned to the LAB species *Coml. paralimentarius* (42.9, 9.0, 33.2, and 24.4% of the total reads for E-B-W, E-B-M, E-B-R, and E-B-S, respectively), *Coml. crustorum* (3.8, 1.1, 0.6, and 1.7%, respectively), *Levl. parabrevis* (2.4, 25.9, 5.8, and 18.2%, respectively), *Lacp. xiangfangensis* (1.4, 11.0, 1.1, and 4.0%, respectively), and *Lentilactobacillus kefiri* (formerly known as *Lactobacillus kefiri*; 0.2, 15.6, 2.4, and 10.4%, respectively). The latter species, as well as *Coml. crustorum*, was not found through microbiological plating, 16S rRNA-PCR-DGGE community profiling, or metagenetic analysis.

The other LAB species mentioned above were shown culture-dependently and/or culture- independently. Still other LAB species, such as *Lentilactobacillus diolivorans* (formerly known as *Lactobacillus diolivorans*), *Lentilactobacillus buchneri* (formerly known as *Lactobacillus buchneri*), *Limosilactobacillus pontis* (formerly known as *Lactobacillus pontis*), *Lactiplantibacillus plantarum* subsp. *argentoratensis* (formerly known as *Lactobacillus plantarum* subsp. *argentoratensis*), *Lacp. plantarum* subsp. *plantarum, Lacc. paracasei* subsp. *paracasei, Lacc. paracasei* subsp. *tolerans, Secundilactobacillus pentosiphilus* (formerly known as *Lactobacillus pentosiphilus*), *Furfurilactobacillus rossiae* (formerly known as *Lactobacillus rossiae*), *Levilactobacillus spicheri* (formerly known as *Lactobacillus spicheri*) and *Lactobacillus helveticus*, were found in at least one of the four sourdough samples analyzed. *Fructilactobacillus sanfranciscensis* was not found through shotgun metagenomics, confirming the culture-dependent data. The AAB species diversity found through metagenomic recruitment plotting was more diverse than what was found through culture-dependent analysis, 16S rRNA-PCR-DGGE community profiling, and metagenetics. Indeed, *A. oryzifermentans* (5.1, 0.5, 0.5, and 0.4% of the total reads for sourdough samples E-B-W, E-B-M, E-B-R, and E-B-S, respectively), *A. pasteurianus* (1.8, 3.0, 2.4, and 4.4%, respectively), *K. sucrofermentans* (0.5, 0.5, 0.2, and 0.3%, respectively), and *Komagataeibacter nataicola* (0.1% in all samples) were found in the four sourdough samples. In all sourdough samples of bakery producer E, except for sourdough sample E-B-R, *A. malorum* (4.1, 1.0, and 1.0% for sourdough samples E-B-W, E-B-M, and E-B-S, respectively), *Acetobacter tropicalis* (0.2, 0.1, and 0.1%, respectively), *A. cerevisiae* (0.7, 0.2, and 0.2%, respectively), *A. sicerae* (0.2, 0.4, and 0.1%, respectively), and *Komagataeibacter hansenii* (0.1% in all sourdough samples) were found.

Different *Pantoea* and *Pseudomonas* spp. were also found, albeit at very low read numbers, with *Pantoea agglomerans* (0.07, 0.03, 0.22, and 0.11% of the total reads for sourdough samples E-B-W, E-B-M, E-B-R, and E-B-S, respectively) and *Pseudomonas poae* (0.01, <0.01, 0.03, and 0.01%, respectively) as the most prevalent species of these genera.

A yeast species related to *Kazachstania turicensis* (0.1, 0.6, 0.4, and 0.3% of the total reads for sourdough samples E-B-W, E-B-M, E-B-R, and E-B-S, respectively) was present in the four sourdough samples, albeit representing low read numbers. Instead, culture-dependent analysis retrieved *K. bulderi* as the most prevalent yeast species, but due to a lack of its complete genome in the NCBI database, it could not be included during the metagenomic recruitment plotting analysis. *Saccharomyces cerevisiae* (0.02, 0.01, and 0.01% for sourdough samples E-B-W, E-B-M, and E-B-S, respectively) was found in three of the four sourdough samples. It was not found in sourdough sample E-B-R, confirming the culture-dependent and metagenetic analyses. Reads belonging to a species related to the arbuscular mycorrhizal fungus *Rhizophagus irregularis* were present in the four sourdough samples as well.

### Substrate and Metabolite Profiles

**Figure 7:**
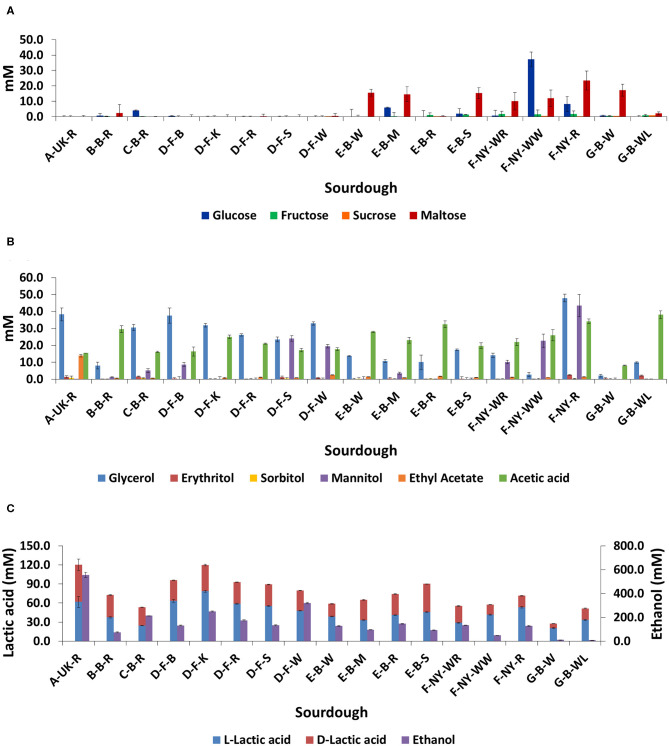
Residual concentrations of glucose, fructose, sucrose, and maltose **(A)**, and the concentrations of glycerol, erythritol, sorbitol, mannitol, ethyl acetate, acetic acid **(B)**, L-lactic acid, D-lactic acid, and ethanol **(C)** in 17 sourdough samples from backslopped sourdoughs of different origins. The sample codes are as described in Table 1.

No residual carbohydrates (glucose, fructose, sucrose, or maltose) were found in the sourdough samples from household producer A and bakery producer D, and only low concentrations in sourdough samples B-B-R, C-B-R, E-B-R, and G-B-WL (Figure 7A). The main carbohydrate, maltose, was still present in sourdough samples E-B-W, E-B-M, E-B-S, G-B-W, and those of bakery producer F (lowest concentration in F-NY-WR and highest concentration in F-NY-R). A high concentration of glucose was still present in sourdough sample F-NY-WW and lower concentrations in sourdough samples C-B-R, E-B-M, and F-NY-R.

The highest concentrations of glycerol and mannitol were found in sourdough sample F-NY-R (47.91 ± 2.28 mM and 43.39 ± 6.56 mM, respectively). Based on a Spearman correlation analysis, these two sugar alcohols positively correlated with the presence of *S. cerevisiae* and/or *Fl. sanfranciscensis* (Figure 5). High concentrations of glycerol were also found in the sourdough samples of household producer A and bakery producers C and D, lower concentrations in those of household producer B and bakery producer E as well as in sourdough samples F-NY-WR and G-B-WL, and glycerol was absent in sourdough samples F-NY-WW and G-B-W (Figure 7B). High concentrations of mannitol were only found in sourdough samples D-F-S, D-F-W, and F-NY-WW and lower ones in D-F-B and F-NY-WR, followed by C-B-R and E-B-M. No or very low concentrations of erythritol and sorbitol were found in all sourdough samples. Ethyl acetate was produced mainly in sourdough sample A-UK-R (13.84 ± 0.75 mM). Acetoin and diacetyl were absent in all sourdough samples. Acetic acid was present in all sourdough samples, with the highest concentration in G-B-WL (38.04 ± 2.34 mM) and the lowest one in G-B-W (8.08 ± 0.19 mM).

Lactic acid was present in all sourdough samples too. The highest concentrations of lactic acid were found in sourdough samples A-UK-R (120.37 ± 17.44 mM) and D-F-K (119.71 ± 2.74 mM); the lowest one in G-B-W (27.52 ± 1.42 mM) (Figure 7C). In all sourdough samples, more L-lactic acid than D-lactic acid occurred, except for sourdough sample C-B-R (45.7% L-lactic acid). The highest ratio of L-lactic acid to D-lactic acid was found in the sourdough samples of bakery producer D (60.1–66.2% L-lactic acid), lambic brewery producer G (64.9–74.9%), as well as in sourdough samples E-B-W (66.3%), F-NY-WW (73.1%), and F-NY-R (74.8%). Ethanol was found in all sourdoughs, with the highest concentration in sourdough sample A-UK-R (555.33 ± 22.06 mM) and the lowest in the sourdoughs of lambic brewery producer G (8.07 ± 0.55 mM in G-B-WL and 10.24 ± 0.27 mM in G-B-W). Moreover, AAB species negatively correlated with the presence of glycerol, ethanol, and to a minor extent lactic acid (both isomers) (Figure 5).

## Discussion

Sourdough ecosystems have been studied intensively in the last few decades, not only to map their microbial community structure but also to assess the influence of the flour and non-flour ingredients, several process parameters, and the region of production on their LAB and yeast species diversity (De Vuyst et al., 2014, 2017; Minervini et al., 2014, 2016; Van Kerrebroeck et al., 2017). However, neither the nature of the flour nor the geographical region of the producers impact the microbial community structure of mature sourdoughs, which was confirmed by the analysis of the sourdoughs examined during the present study (Ercolini et al., 2013; De Vuyst et al., 2014, 2017; Van Kerrebroeck et al., 2017). Although some non-flour ingredients may be responsible for the occurrence of certain LAB or yeast species (Minervini et al., 2016; Ripari et al., 2016b), the dough yield, pH, and temperature are usually of direct impact on the nature of the LAB species present (Di Cagno et al., 2014; De Vuyst et al., 2016, 2017; Van Kerrebroeck et al., 2017). A common feature of sourdoughs is the LAB to yeast ratio of 10:1 to 100:1, which was also the case for most of the sourdoughs analyzed during the present study, as well as the occurrence of *Lactobacillaceae* and ascomycetous yeasts (Gobbetti, 1998; Hammes et al., 2005; De Vuyst et al., 2014, 2017). Yet, the microbial composition of all sourdoughs of the present study was similar when coming from the same producer, which was also independent of the type of flour used when the producer made use of different flours, but most likely underlined the importance of the house microbiota (Minervini et al., 2015) or the process conditions applied (Ercolini et al., 2013). For instance, the sourdoughs of the French artisan bakery producer D, although prepared from different flours, harbored only *Fl. sanfranciscensis* and *S. cerevisiae*, those of the Belgian artisan bakery producer E mainly *Coml. paralimentarius, Lacp. xiangfangensis, Levl. brevis, S. cerevisiae, K. bulderi*, and several AAB species, those of the American artisan bakery producer F mainly *Fl. sanfranciscensis* and *K. humilis*, and those of the Belgian artisan lambic brewery producer G mainly *Lacc. paracasei, D. anomala*, and *Acetobacter* species. It has been shown before that French sourdoughs often contain *Fl. sanfranciscensis* (Ferchichi et al., 2007, 2008; Robert et al., 2009; Lhomme et al., 2014, 2015a,b, 2016; Michel et al., 2016) and that artisan sourdoughs may contain either a restricted or a wide microbial consortium, whether or not depending on the house microbiota (Scheirlinck et al., 2009; Minervini et al., 2015). The presence of *D. anomala* and diverse AAB species in the sourdoughs of the artisan lambic brewery producer G most likely originated from the lambic beer or brewery environment (Spitaels et al., 2014a,b,c, 2015, 2017; De Roos and De Vuyst, 2018, 2019; De Roos et al., 2018a).

*Levilactobacillus brevis* and *Lacp. plantarum* were detected culture-dependently and/or culture-independently in all household sourdoughs examined, showing a possible adaptation of this species to the household environment, as it has been found in household sourdoughs of different origins before (Gänzle and Zheng, 2019). The backslopped household sourdough of United Kingdom origin harbored not only other different sourdough-common microorganisms, such as *S. cerevisiae*, but also the sourdough-uncommon LAB species *Lacc. paracasei* subsp. *tolerans/paracasei*. Moreover, *Lacc. paracasei* subsp. *paracasei* was prevalent in the lambic brewery sourdoughs. However, this LAB species is not typical for sourdough nor for lambic beer production (De Vuyst et al., 2017; De Roos and De Vuyst, 2019). Its active growth was likely reflected in the high percentage of the L-lactate enantiomer, which is typical for this dairy LAB species (Maragkoudakis et al., 2006; Moon et al., 2012). In general, differences in the lactic acid enantiomer ratio depended on the LAB species present in the sourdoughs (Dicks and Endo, 2009). For instance, the growth of *Coml. crustorum* is also responsible for a high production of the L-lactate enantiomer (Scheirlinck et al., 2007; Comasio et al., 2019). Also the occurrence of *Fl. sanfranciscensis* is responsible for a high production of L-lactic acid (Maruyama and Okada, 2006).

Species of the *Levilactobacillus* genus (formerly the *Lactobacillus brevis* group) were isolated from household sourdoughs solely, although they commonly occur in backslopped sourdoughs (De Vuyst et al., 2017). One of the Belgian sourdoughs from household origin harbored the sourdough-uncommon *P. parvulus* (likely responsible for the high production of D-lactic acid) and *Pi. fermentans* as the prevailing LAB and yeast species, respectively. Both microbial species are usually associated with other fermented food matrices. Although *Pi. fermentans* has been isolated once from an artisan bakery sourdough, this yeast species is mainly found in cheese and as contaminant in fruit juices (Arias et al., 2002; Succi et al., 2003; Kurtzman et al., 2011). In contrast, *P. parvulus* has never been found in sourdoughs; this LAB species is mainly retrieved as contaminant from minimally processed vegetables and fermented beverages, such as cider and wine (Davis et al., 1986; Fernández et al., 1996; Bennik et al., 1997).

AAB species were found only in the Belgian sourdoughs. This may be associated with cross-contamination in the production places examined, as the artisan bakery involved uses a lot of fruits in the production room and the lambic brewery obviously houses AAB (De Vuyst et al., 2017; De Roos and De Vuyst, 2018, 2019). Also, fruit flies may be more present in the bakery and act as carrier of AAB. Fruits are a natural habitat of several AAB species (Matsushita et al., 2016). Lambic beer is a source of AAB (Spitaels et al., 2014a,b,c, 2015, 2017; De Roos and De Vuyst, 2018, 2019; De Roos et al., 2018a). Moreover, the lowest concentrations of ethanol and acetic acid were present in the brewery sourdough with the highest number of AAB, whose species not only grew but also oxidized ethanol to acetic acid that was further over-oxidized to carbon dioxide and water. *Acetobacter* species can oxidize acetic acid through the tricarboxylic acid cycle, but only when ethanol is completely depleted (De Roos and De Vuyst, 2019). Also, glycerol (that was absent in this brewery sourdough) can be oxidized by AAB to dihydroxyacetone phosphate (when ethanol is depleted), which can be further converted into acetic acid and carbon dioxide *via* the Embden-Meyerhof-Parnas pathway and gluconeogenesis (Mamlouk and Gullo, 2013; Komagata et al., 2014; Matsushita et al., 2016). Further, *D. anomala* was the prevailing yeast species in the lambic brewery sourdoughs, at least as shown culture-dependently, whereas 26S rRNA-PCR-DGGE community profiling identified *D. bruxellensis*, indicating survival and activity of these lambic beer yeasts in the lambic brewery sourdoughs. Both *Dekkera* species are indeed typical for the maturation of lambic beer (De Roos and De Vuyst, 2019). *Dekkera bruxellensis* was detected before through 26S rRNA-PCR-DGGE community profiling in a rye sourdough made with a mixed starter culture, encompassing different LAB species, *K. humilis* and *S. cerevisiae*, even after several backslopping steps (Meroth et al., 2003). *Dekkera anomala* was never found in a spontaneously fermented sourdough up to now. The present study showed its possible adaptation to a sourdough matrix, at least under the environmental and fermentation conditions applied. Although not monitored, *D. anomala* has been used as non-conventional yeast in dough making, causing a low carbon dioxide production because of a slow metabolism or its non-survival (Aslankoohi et al., 2016). Finally, *Pi. membranifaciens* could be retrieved only by amplicon-based metagenetic sequencing, although it is also present during lambic beer maturation (Spitaels et al., 2014c).

The sourdough-specific LAB species *Fl. sanfranciscensis* occurred in the French and American sourdoughs analyzed, in association with *S. cerevisiae* and *K. humilis*, respectively. This strictly heterofermentative LAB species uses fructose as alternative external electron acceptor, converting it into mannitol, as reflected in the high concentrations of the latter sugar alcohol found in those sourdoughs (Vogel et al., 2011). Its occurrence may be linked with the presence of insects, as it has been isolated from the insect gut and insect frass that may contaminate stored cereals (Ripari et al., 2016b; Boiocchi et al., 2017), provided the right technological conditions are applied for sourdough making, such as fast backslopping procedures, ambient temperature, and moderate acidic pH (De Vuyst et al., 2017). Moreover, *Fl. sanfranciscensis* is able to survive in sourdoughs made from different flours, as it has been shown in, not only wheat, rye, buckwheat, and teff sourdoughs (De Vuyst et al., 2017), but also in a gluten-free sourdough (prepared from a mixture of buckwheat, rice, and whole-meal rice flours) that was initiated from a wheat sourdough (Lhomme et al., 2014). The sourdoughs of the present study that were dominated by *Fl. sanfranciscensis* displayed lower bacterial diversities than the other sourdoughs examined, confirming meta-analysis data on the occurrence of this sourdough-specific LAB species (Van Kerrebroeck et al., 2017). Furthermore, in sourdoughs dominated by *Fl. sanfranciscensis*, the yeasts *K. humilis* or *S. cerevisiae* were prevalent, confirming the stable association between this maltose-positive LAB species and the maltose-negative *K. humilis* as well the possible co-existence of both the maltose-positive *Fl. sanfranciscensis* and the maltose-positive *S. cerevisiae* (De Vuyst et al., 2014, 2016, 2017; Vigentini et al., 2014; Van Kerrebroeck et al., 2017).

The sourdoughs of the Belgian artisan bakery displayed a rather wide LAB, AAB, and yeast species diversity, which may be due to cross-contamination, as mentioned above for the AAB species. However, the sourdough based on rye differed from these other bakery sourdoughs in that *S. cerevisiae* and some LAB and AAB species as well as residual carbohydrates were absent, whereas the acetic acid concentration was the highest. This may indicate an influence of the rye flour used.

Finally, the present study showed that different techniques used to unravel the microbial composition of different sourdough samples may help to find out their actual taxonomic structure, as they can be both confirmative and complementary. For instance, the use of both rRNA-PCR-DGGE community profiling and metagenetics to analyze the microbial composition of sourdough samples showed that the latter technique detected more species than the former one and microbiological plating, as subdominant species can be detected, sometimes quantitatively (Ercolini, 2013; Viiard et al., 2016). Moreover, whereas AAB species can often hardly be distinguished through rRNA-PCR-DGGE community profiling (Papalexandratou et al., 2011), different AAB species could be identified through metagenetics, in most cases confirming the culture-dependent analysis. However, due to the limited lengths of the DNA fragments amplified as well as the specificity of the DNA region targeted for amplification, species identification was not always possible. Indeed, multiple species were often found after BLAST analysis of DNA fragments obtained through rRNA-PCR-DGGE analysis, making it difficult to distinguish closely related LAB species (e.g., *Coml. paralimentarius* and *Coml. alimentarius, P. parvulus* and *P. inopinatus*). Also, 26S rRNA-PCR-DGGE community profiling based on amplification of the D1 region of the fungal 26S rRNA gene, colony identification based on amplification of the ITS region of the fungal rRNA transcribed unit, and metagenetics based on the ITS1 region could not uniformly identify *S. cerevisiae* or *S. cariocanus* due to their close relatedness (Kurtzman et al., 2011). Further, metagenetics and metagenomics unraveled the presence of more or different species, as these techniques may include subdominant background microorganisms and non-cultured microorganisms, thanks to the use of different taxonomy analysis tools. To the best of our knowledge, this was the first study that applied shotgun metagenomics on sourdough samples. Of particular help was the taxonomical analysis of the metagenomic sequence data, based on metagenomic recruitment plotting of the whole genome of a particular species, which allows also the detection of species that were not found with the other techniques (e.g., *Lenl. kefiri* in the four Belgian artisan bakery sourdoughs) and even new species, such as *Oenococcus sicerae* in water kefir (Verce et al., 2019, 2020). Yet, the finding of *A. tropicalis* through metagenomic recruitment plotting was not in accordance with the culture-dependently isolated and identified *A. senegalensis* (both 16S rRNA and *dna*K gene sequencing). However, both species differ only 4 and 10 nucleotides in their 16S rRNA and *dna*K genes, respectively (this study; Li et al., 2014). Hence, the present study showed not only the complementarity of the combined use of culture-dependent and culture-independent methods regarding species detection and identification, which has been commonly employed since more than 20 years, but also the provision of both qualitative and quantitative data regarding species distribution. Moreover, shotgun metagenomic sequencing gives a deeper insight into the cultivable minor and non-cultivable species diversity, in particular when metagenomic recruitment plotting is applied. Yet, amplicon-based sequencing approaches are straightforward when an ASV instead of an operational taxonomic unit (OTU) approach is applied, as the latter is not always able to detect down to species level (Callahan et al., 2017). Furthermore, relative to metagenetic and DGGE analyses, shotgun metagenomic sequencing is of utmost importance for further inclusion in multiphasic approaches.

In conclusion, the present study showed that the producers of the sourdoughs were the main factors to drive the species diversity, which was mainly reflected in their house microbiota and likely in the process parameters applied, although different relative abundances were found with different flours used by producers that made use of different flours. The combined use of different culture-independent approaches showed the advantage of metagenomics to study sourdough ecosystems. Moreover, the decreasing prices for sequencing and the growing availability of bioinformatics tools and databanks further merits the incorporation of shotgun metagenomics into groundbreaking research on the microbial composition of (spontaneously) fermented foods and beverages.

## Data Availability Statement

The datasets generated for this study can be found in the ENA/EBI: PRJEB35796.

## Author Contributions

AC contributed to the experimental work, the acquisition, processing, interpretation of the data, and drafting of the manuscript. MV contributed in the metagenomic analysis. SV reviewed and edited the manuscript. LD supervised the work, interpreted the data, reviewed, and edited the manuscript. All authors contributed to the article and approved the submitted version.

## Conflict of Interest

The authors declare that the research was conducted in the absence of any commercial or financial relationships that could be construed as a potential conflict of interest.

## Supplementary Material

Supplementary Table 1 Percentages of metagenomic sequence reads from the four metagenomes recruited by the reference genomes of species included in the analysis. Only species considered as present, absent, or absent but having a closely related unknown species present in the metagenomes are mentioned.

## References

[B1] Altschul S. F. Gish W. Miller W. Myers E. W. Lipman D. J. (1990). Basic local alignment search tool. J. Mol. Biol. 215, 403–410. 10.1016/S0022-2836(05)80360-2 2231712

[B2] Andrews S. (2012). FastQC: A Quality Control Tool for High Throughput Sequence Data. Available online at: http://www.bioinformatics.babraham.ac.uk/projects/fastqc (accessed April 1, 2019).

[B3] Arias C. R. Burns J. K. Friedrich L. M. Goodrich R. M. Parish M. E. (2002). Yeast species associated with orange juice: evaluation of different identification methods. Appl. Environ. Microbiol. 68, 1955–1961. 10.1128/AEM.68.4.1955-1961.2002 11916718 PMC123878

[B4] Aslankoohi E. Herrera-Malaver B. Rezaei M. N. Steensels J. Courtin C. M. Verstrepen K. J. (2016). Non-conventional yeast strains increase the aroma complexity of bread. PLoS ONE. 11:e0165126. 10.1371/journal.pone.0165126 27776154 PMC5077118

[B5] Bennik M. Smid E. Gorris L. (1997). Vegetable-associated *Pediococcus parvulus* produces pediocin PA-1. Appl. Environ. Microbiol. 63, 2074–2076. 10.1128/AEM.63.5.2074-2076.1997 16535615 PMC1389170

[B6] Boiocchi F. Porcellato D. Limonta L. Picozzi C. Vigentini I. Locatelli D. P.. (2017). Insect frass in stored cereal products as a potential source of *Lactobacillus sanfranciscensis* for sourdough ecosystem. J. Appl. Microbiol. 123, 944–955. 10.1111/jam.13546 28736890

[B7] Bokulich N. A. Mills D. A. (2013). Improved selection of internal transcribed spacer-specific primers enables quantitative, ultra-high-throughput profiling of fungal communities. Appl. Environ. Microbiol. 79, 2519–2526. 10.1128/AEM.03870-12 23377949 PMC3623200

[B8] Buchfink B. Xie C. Huson D. H. (2015). Fast and sensitive protein alignment using DIAMOND. Nat. Methods 12, 59–60. 10.1038/nmeth.3176 25402007

[B9] Callahan B. J. McMurdie P. J. Holmes S. P. (2017). Exact sequence variants should replace operational taxonomic units in marker-gene data analysis. ISME J. 11, 2639–2643. 10.1038/ismej.2017.119 28731476 PMC5702726

[B10] Caporaso J. G. Lauber C. L. Walters W. A. Berg-Lyons D. Lozupone C. A. Turnbaugh P. J.. (2011). Global patterns of 16S rRNA diversity at a depth of millions of sequences per sample. Proc. Natl. Acad. Sci. U.S.A. 108, 4516–4522. 10.1073/pnas.1000080107 20534432 PMC3063599

[B11] Cleenwerck I. De Vos P. De Vuyst L. (2010). Phylogeny and differentiation of species of the genus *Gluconacetobacter* and related taxa based on multilocus sequence analyses of housekeeping genes and reclassification of *Acetobacter xylinus* subsp. *sucrofermentans* as *Gluconacetobacter sucrofermentans* (Toyosaki et al., 1996) sp. nov., comb. nov. Int. J. Syst. Evol. Microbiol. 60, 2277–2283. 10.1099/ijs.0.018465-0 19915110

[B12] Cocolin L. Bisson L. F. Mills D. A. (2000). Direct profiling of the yeast dynamics in wine fermentations. FEMS Microbiol. Lett. 189, 81–87. 10.1111/j.1574-6968.2000.tb09210.x 10913870

[B13] Coda R. Di Cagno R. Gobbetti M. Rizzello C. G. (2014). Sourdough lactic acid bacteria: exploration of non-wheat cereal-based fermentation. Food Microbiol. 37, 51–58. 10.1016/j.fm.2013.06.018 24230473

[B14] Comasio A. Harth H. Weckx S. De Vuyst L. (2019). The addition of citrate stimulates the production of acetoin and diacetyl by a citrate-positive *Lactobacillus crustorum* strain during wheat sourdough fermentation. Int. J. Food Microbiol. 289, 88–105. 10.1016/j.ijfoodmicro.2018.08.030 30218873

[B15] Davis C. R. Wibowo D. Fleet G. H. Lee T. H. (1986). Growth and metabolism of lactic acid bacteria during and after malolactic fermentation of wines at different pH. Appl. Environ. Microbiol. 51, 539–545. 10.1128/AEM.51.3.539-545.1986 16347015 PMC238915

[B16] De Bruyn F. Zhang S. J. Pothakos V. Torres J. Lambot C. Moroni A. V.. (2017). Exploring the impacts of postharvest processing on the microbiota and metabolite profiles during green co?ee bean production. Appl. Environ. Microbiol. 83, e02398–e02316. 10.1128/AEM.02398-16 27793826 PMC5165123

[B17] De Roos J. De Vuyst L. (2018). Acetic acid bacteria in fermented foods and beverages. Curr. Opin. Biotechnol. 49, 115–119. 10.1016/j.copbio.2017.08.007 28863341

[B18] De Roos J. De Vuyst L. (2019). Microbial acidification, alcoholization, and aroma production during spontaneous lambic beer production. J. Sci. Food Agric. 99, 25–38. 10.1002/jsfa.9291 30246252

[B19] De Roos J. Vandamme P. De Vuyst L. (2018b). Wort substrate consumption and metabolite production during lambic beer fermentation and maturation explain the successive growth of specific bacterial and yeast species. Front. Microbiol. 9:2763. 10.3389/fmicb.2018.02763 30510547 PMC6252343

[B20] De Roos J. Verce M. Aerts M. Vandamme P. De Vuyst L. (2018a). Temporal and spatial distribution of the acetic acid bacterium communities throughout the wooden casks used for the fermentation and maturation of lambic beer underlines their functional role. Appl. Environ. Microbiol. 84, e02846–e02817. 10.1128/AEM.02846-17 29352086 PMC5861831

[B21] De Vuyst L. Harth H. van Kerrebroeck S. Leroy F. (2016). Yeast diversity of sourdoughs and associated metabolic properties and functionalities. Int. J. Food Microbiol. 239, 26–34. 10.1016/j.ijfoodmicro.2016.07.018 27470533

[B22] De Vuyst L. van Kerrebroeck S. Harth H. Huys G. Daniel H.-M. Weckx S. (2014). Microbial ecology of sourdough fermentations: diverse or uniform? Food Microbiol. 37, 11–29. 10.1016/j.fm.2013.06.002 24230469

[B23] De Vuyst L. van Kerrebroeck S. Leroy F. (2017). Microbial ecology and process technology of sourdough fermentation. Adv. Appl. Microbiol. 100, 49–160. 10.1016/bs.aambs.2017.02.003 28732554

[B24] Di Cagno R. Pontonio E. Buchin S. De Angelis M. Lattanzi A. Valerio F.. (2014). Diversity of the lactic acid bacterium and yeast microbiota in the switch from firm- to liquid-sourdough fermentation. Appl. Environ. Microbiol. 80, 3161–3172. 10.1128/AEM.00309-14 24632249 PMC4018931

[B25] Dicks L. M. T. Endo A. (2009). Taxonomic status of lactic acid bacteria in wine and key characteristics to differentiate species. S. Afr. J. Enol. Vitic. 30, 72–90. 10.21548/30-1-1427

[B26] Edwards U. Rogall T. Blöcker H. Emde M. Böttger E. C. (1989). Isolation and direct complete nucleotide determination of entire genes. Characterization of a gene coding for 16S ribosomal RNA. Nucleic Acids Res. 17, 7843–7853. 10.1093/nar/17.19.7843 2798131 PMC334891

[B27] Ercolini D. (2013). High-throughput sequencing and metagenomics: moving forward in the culture-independent analysis of food microbial ecology. Appl. Environ. Microbiol. 79, 3148–3155. 10.1128/AEM.00256-13 23475615 PMC3685257

[B28] Ercolini D. Pontonio E. De Filippis F. Minervini F. La Storia A. Gobbetti M.. (2013). Microbial ecology dynamics during rye and wheat sourdough preparation. Appl. Environ. Microbiol. 79, 7827–7836. 10.1128/AEM.02955-13 24096427 PMC3837820

[B29] Ferchichi M. Valcheva R. Oheix N. Kabadjova P. Prévost H. Onno B. (2008). Rapid investigation of French sourdough microbiota by restriction fragment length polymorphism of the 16S-23S rRNA gene intergenic spacer region. World J. Microbiol. Biotechnol. 24, 2425–2434. 10.1007/s11274-008-9763-x

[B30] Ferchichi M. Valcheva R. Prévost H. Onno B. Dousset X. (2007). Molecular identification of the microbiota of French sourdough using temporal temperature gradient gel electrophoresis. Food Microbiol. 24, 678–686. 10.1016/j.fm.2007.04.001 17613364

[B31] Fernández K. Dueñas M. Irastorza A. Bilbao A. del Campo G. (1996). Characterization and DNA plasmid analysis of ropy *Pediococcus* spp. strains isolated from Basque country ciders. J. Food Protect. 59, 35–40. 10.4315/0362-028X-59.1.35 31158968

[B32] Gänzle M. G. Ehmann M. Hammes W. P. (1998). Modelling of growth of *Lactobacillus sanfranciscensis* and *Candida milleri* in response to process parameters of the sourdough fermentation. Appl. Environ. Microbiol. 64, 2616–2623. 10.1128/AEM.64.7.2616-2623.1998 9647838 PMC106434

[B33] Gänzle M. G. Ripari V. (2016). Composition and function of sourdough microbiota: from ecological theory to bread quality. Int. J. Food Microbiol. 239, 19–25. 10.1016/j.ijfoodmicro.2016.05.004 27240932

[B34] Gänzle M. G. Zheng J. (2019). Lifestyles of sourdough lactobacilli - do they matter for microbial ecology and bread quality? Int. J. Food Microbiol. 302, 15–23. 10.1016/j.ijfoodmicro.2018.08.019 30172443

[B35] Gevers D. Huys G. Swings J. (2001). Applicability of rep-PCR fingerprinting for identification of *Lactobacillus* species. FEMS Microbiol. Lett. 205, 31–36. 10.1111/j.1574-6968.2001.tb10921.x 11728712

[B36] Gobbetti M. (1998). The sourdough microflora: interactions between lactic acid bacteria and yeasts in sourdoughs. Trends Food Sci. Technol. 9, 267–274. 10.1016/S0924-2244(98)00053-3 24421010

[B37] Gobbetti M. De Angelis M. Di Cagno R. Calasso M. Archetti G. Rizzello C. G. (2019). Novel insights on the functional/nutritional features of the sourdough fermentation. Int. J. Food Microbiol. 302, 103–113. 10.1016/j.ijfoodmicro.2018.05.018 29801967

[B38] Guerzoni M. E. Vernocchi P. Ndagijimana M. Gianotti A. Lanciotti R. (2007). Generation of aroma compounds in sourdough: effects of stress exposure and lactobacilli-yeasts interactions. Food Microbiol. 24, 139–148. 10.1016/j.fm.2006.07.007 17008156

[B39] Hammes W. P. Brandt M. J. Francis K. L. Rosenheim M. Seitter F. H. Vogelmann S. (2005). Microbial ecology of cereal fermentations. Trends Food Sci. Technol. 16, 4–11. 10.1016/j.tifs.2004.02.010

[B40] Harrell F. E. J. (2018). Hmisc: Harrell Miscellaneous. R package version 4.1.1. Available online at: https://cran.r-project.org/web/packages/Hmisc (accessed April 1, 2019).

[B41] Harth H. van Kerrebroeck S. De Vuyst L. (2016). Community dynamics and metabolite target analysis of spontaneous, backslopped barley sourdough fermentations under laboratory and bakery conditions. Int. J. Food Microbiol. 228, 22–32. 10.1016/j.ijfoodmicro.2016.04.011 27088869

[B42] Hermann M. Petermeier H. Vogel R. F. (2015). Development of novel sourdoughs with in *situ* formed exopolysaccharides from acetic acid bacteria. Eur. Food Res. Technol. 241, 185–197. 10.1007/s00217-015-2444-8

[B43] Huson D. H. Beier S. Flade I. Górska A. El-Hadidi M. Mitra S.. (2016). MEGAN community edition - interactive exploration and analysis of large-scale microbiome sequencing data. PLoS Comput. Biol. 12:e1004957. 10.1371/journal.pcbi.1004957 27327495 PMC4915700

[B44] Jacques N. Sarilar V. Urien C. Lopes M. R. Morais C. G. Uetanabaro A. P. T.. (2016). Three novel ascomycetous yeast species of the *Kazachstania* clade, *Kazachstania saulgeensis* sp. nov., *Kazachstania serrabonitensis* sp. nov. and *Kazachstania australis* sp. nov. reassignment of *Candida humilis* to *Kazachstania humilis* f.a. comb. nov. and *Candida pseudohumilis* to *Kazachstania pseudohumilis* f.a. comb. nov. Int. J. Syst. Evol. Microbiol. 66, 5192–5200. 10.1099/ijsem.0.001495 27902197

[B45] Kassambara A. Mundt F. (2020). Factoextra: Extract and Visualize the Results of Multivariate Data Analyses. R package version 1.0.7. Available online at: https://CRAN.R-project.org/package=factoextra (accessed April 20, 2020).

[B46] Kõljalg U. Larsson K. H. Abarenkov K. Nilsson R. H. Alexander I. J. Eberhardt U.. (2005). UNITE: a database providing web-based methods for the molecular identification of ectomycorrhizal fungi. New Phytol. 166, 1063–1068. 10.1111/j.1469-8137.2005.01376.x 15869663

[B47] Komagata K. Iino T. Yamada Y. (2014). The family Acetobacteraceae, in The Prokaryotes. Alphaproteobacteria and Betaproteobacteria, eds Rosenberg E. De Long E. F. Lory S. Stackebrandt E. Thompson F. (Berlin: Springer), 3–78.

[B48] Kurtzman C. P. Fell J. W. Boekhout T. (2011). The Yeasts: A Taxonomic Study. Amsterdam: Elsevier.

[B49] Laureys D. De Vuyst L. (2014). Microbial species diversity, community dynamics, and metabolite kinetics of water kefir fermentation. Appl. Environ. Microbiol. 80, 2564–2572. 10.1128/AEM.03978-13 24532061 PMC3993195

[B50] Lhomme E. Lattanzi A. Dousset X. Minervini F. De Angelis M. Lacaze G.. (2015a). Lactic acid bacterium and yeast microbiotas of sixteen French traditional sourdoughs. Int. J. Food Microbiol. 215, 161–170. 10.1016/j.ijfoodmicro.2015.09.015 26439422

[B51] Lhomme E. Mezaize S. Ducasse M. B. Chiron H. Champomier-Verges M. C. Chaillou S.. (2014). A polyphasic approach to study the dynamics of microbial population of an organic wheat sourdough during its conversion to gluten-free sourdough. Int. Microbiol. 17, 1–9. 10.2436/20.1501.01.202 25296441

[B52] Lhomme E. Onno B. Chuat V. Durand K. Orain S. Valence F.. (2016). Genotypic diversity of *Lactobacillus sanfranciscensis* strains isolated from French organic sourdoughs. Int. J. Food Microbiol. 226, 13–19. 10.1016/j.ijfoodmicro.2016.03.008 27015297

[B53] Lhomme E. Orain S. Courcoux P. Onno B. Dousset X. (2015b). The predominance of *Lactobacillus sanfranciscensis* in French organic sourdoughs and its impact on related bread characteristics. Int. J. Food Microbiol. 213, 40–48. 10.1016/j.ijfoodmicro.2015.05.010 26051957

[B54] Li L. Wieme A. Spitaels F. Balzarini T. Nunes O. G. Manaia C. M.. (2014). *Acetobacter sicerae* sp. *nov.*, isolated from cider and kefir, and identification of species of the genus Acetobacter by dnaK, groEL and rpoB sequence analysis. Int. J. Syst. Evol. Microbiol. 64, 2407–2415. 10.1099/ijs.0.058354-0 24763601

[B55] Li Z. Li H. Bian K. (2016). Microbiological characterization of traditional dough fermentation starter (jiaozi) for steamed bread making by culture-dependent and culture-independent methods. Int. J. Food Microbiol. 234, 9–14. 10.1016/j.ijfoodmicro.2016.06.024 27351835

[B56] Liu T. Li Y. Chen J. Sadiq F. A. Zhang G. Li Y. (2016). Prevalence and diversity of lactic acid bacteria in Chinese traditional sourdough revealed by culture dependent and pyrosequencing approaches. Food Sci. Technol. 68, 91–97. 10.1016/j.lwt.2015.12.025

[B57] Mamlouk D. Gullo M. (2013). Acetic acid bacteria: physiology and carbon sources oxidation. Indian J. Microbiol. 53, 377–384. 10.1007/s12088-013-0414-z 24426139 PMC3779290

[B58] Maragkoudakis P. A. Zoumpopoulou G. Miaris C. Kalantzopoulos G. Pot B. Tsakalidou E. (2006). Probiotic potential of *Lactobacillus* strains isolated from dairy products. Int. Dairy J. 16, 189–199. 10.1016/j.idairyj.2005.02.009

[B59] Martin M. (2011). Cutadapt removes adapter sequences from high-throughput sequencing reads. EMBnet J. 17, 10–12. 10.14806/ej.17.1.200

[B60] Maruyama Y. Okada S. (2006). Microorganisms involved in the fermentation process of panettone sourdough maintaining in Japan. Food Preserv. Sci. 32, 59–66. 10.5891/jafps.32.59

[B61] Matsushita K. Toyama H. Tonouchi N. Okamoto-Kainuma A. (2016). Acetic Acid Bacteria: Ecology and Physiology. Tokyo: Springer.

[B62] Menzel P. Ng K. L. Krogh A. (2016). Fast and sensitive taxonomic classification for metagenomics with Kaiju. Nat. Commun. 7:11257. 10.1038/ncomms11257 27071849 PMC4833860

[B63] Meroth C. B. Hammes W. P. Hertel C. (2003). Identification and population dynamics of yeasts in sourdough fermentation processes by PCR-denaturing gradient gel electrophoresis. Appl. Environ. Microbiol. 69, 7453–7461. 10.1128/AEM.69.12.7453-7461.2003 14660398 PMC309968

[B64] Michel E. Monfort C. Deffrasnes M. Guezenec S. Lhomme E. Barret M.. (2016). Characterization of relative abundance of lactic acid bacteria species in French organic sourdough by cultural, qPCR and MiSeq high-throughput sequencing methods. Int. J. Food Microbiol. 239, 35–43. 10.1016/j.ijfoodmicro.2016.07.034 27539249

[B65] Minervini F. Celano G. Lattanzi A. De Angelis M. Gobbetti M. (2016). Added ingredients affect the microbiota and biochemical characteristics of durum wheat type-I sourdough. Food Microbiol. 60, 112–123. 10.1016/j.fm.2016.05.016 27554152

[B66] Minervini F. De Angelis M. Di Cagno R. Gobbetti M. (2014). Ecological parameters influencing microbial diversity and stability of traditional sourdough. Int. J. Food Microbiol. 171, 136–146. 10.1016/j.ijfoodmicro.2013.11.021 24355817

[B67] Minervini F. Di Cagno R. Lattanzi A. De Angelis M. Antonielli L. Cardinali G. (2012a). Lactic acid bacterium and yeast microbiotas of 19 sourdoughs used for traditional/typical Italian breads: interactions between ingredients and microbial species diversity. Appl. Environ. Microbiol. 78, 1251–1264. 10.1128/AEM.07721-11 22156414 PMC3273004

[B68] Minervini F. Lattanzi A. De Angelis M. Celano G. Gobbetti M. (2015). House microbiotas as sources of lactic acid bacteria and yeasts in traditional Italian sourdoughs. Food Microbiol. 52, 66–76. 10.1016/j.fm.2015.06.009 26338118

[B69] Minervini F. Lattanzi A. De Angelis M. Di Cagno R. Gobbetti M. (2012b). Influence of artisan bakery- or laboratory-propagated sourdoughs on the diversity of lactic acid bacterium and yeast microbiotas. Appl. Environ. Microbiol. 78, 5328–5340. 10.1128/AEM.00572-12 22635989 PMC3416412

[B70] Moens F. Lefeber T. De Vuyst L. (2014). Oxidation of metabolites highlights the microbial interactions and role of *Acetobacter pasteurianus* during cocoa bean fermentation. Appl. Environ. Microbiol. 80, 1848–1857. 10.1128/AEM.03344-13 24413595 PMC3957632

[B71] Moon S. K. Wee Y. J. Choi G. W. (2012). A novel lactic acid bacterium for the production of high purity L-lactic acid, *Lactobacillus paracasei* subsp. *paracasei* CHB2121. J. Biosci. Bioeng. 114, 155–159. 10.1016/j.jbiosc.2012.03.016 22578598

[B72] Muyzer G. De Waal E. C. Uitterlinden A. G. (1993). Profiling of complex microbial populations by denaturing gradient gel electrophoresis analysis of polymerase chain reaction-amplified genes coding for 16S rRNA. Appl. Environ. Microbiol. 59, 695–700. 10.1128/AEM.59.3.695-700.1993 7683183 PMC202176

[B73] Palla M. Cristani C. Giovannetti M. Agnolucci M. (2017). Identification and characterization of lactic acid bacteria and yeasts of PDO Tuscan bread sourdough by culture dependent and independent methods. Int. J. Food Microbiol. 250, 19–26. 10.1016/j.ijfoodmicro.2017.03.015 28364622

[B74] Papalexandratou Z. Falony G. Romanens E. Jimenez J. C. Amores F. Daniel H.-M.. (2011). Species diversity, community dynamics, and metabolite kinetics of the microbiota associated with traditional Ecuadorian spontaneous cocoa bean fermentations. Appl. Environ. Microbiol. 77, 7698–7714. 10.1128/AEM.05523-11 21926224 PMC3209185

[B75] Papalexandratou Z. Lefeber T. Bahrim B. Lee O. Daniel H.-M. De Vuyst L. (2013). *Hanseniaspora opuntiae, Saccharomyces cerevisiae, Lactobacillus fermentum*, and *Acetobacter pasteurianus* predominate during well-performed Malaysian cocoa bean box fermentations, underlining the importance of these microbial species for a successful cocoa bean fermentation process. Food Microbiol. 35, 73–85. 10.1016/j.fm.2013.02.015 23664257

[B76] Quast C. Pruesse E. Yilmaz P. Gerken J. Schweer T. Yarza P.. (2013). The SILVA ribosomal RNA gene database project: improved data processing and web-based tools. Nucleic Acids Res. 41, D590–D596. 10.1093/nar/gks1219 23193283 PMC3531112

[B77] R Core Team (2017). R: A Language and Environment for Statistical Computing. R Foundation for Statistical Computing, Vienna. Available online at: https://www.R-project.org/ (accessed April 20, 2020).

[B78] Ricciardi A. Parente E. Piraino P. Paraggio M. Romano P. (2005). Phenotypic characterization of lactic acid bacteria from sourdoughs for Altamura bread produced in Apulia (Southern Italy). Int. J. Food Microbiol. 98, 63–72. 10.1016/j.ijfoodmicro.2004.05.007 15617801

[B79] Ripari V. Cecchi T. Berardi E. (2016a). Microbiological characterisation and volatiles profile of model, *ex-novo*, and traditional Italian white wheat sourdoughs. Food Chem. 205, 297–307. 10.1016/j.foodchem.2016.02.150 27006243

[B80] Ripari V. Gänzle M. G. Berardi E. (2016b). Evolution of sourdough microbiota in spontaneous sourdoughs started with different plant materials. Int. J. Food Microbiol. 232, 35–42. 10.1016/j.ijfoodmicro.2016.05.025 27240218

[B81] Robert H. Gabriel V. Fontagné-Faucher C. (2009). Biodiversity of lactic acid bacteria in French wheat sourdough as determined by molecular characterization using species-specific PCR. Int. J. Food Microbiol. 135, 53–59. 10.1016/j.ijfoodmicro.2009.07.006 19651455

[B82] RStudio Team (2015). RStudio: Integrated Development for R. RStudio, Inc. Boston, MA. Available online at: http://www.rstudio.com (accessed April 20, 2020).

[B83] Scheirlinck I. van der Meulen R. De Vuyst L. Vandamme P. Huys G. (2009). Molecular source tracking of predominant lactic acid bacteria in traditional Belgian sourdoughs and their production environments. J. Appl. Microbiol. 106, 1081–1092. 10.1111/j.1365-2672.2008.04094.x 19187144

[B84] Scheirlinck I. van der Meulen R. van Schoor A. Huys G. Vandamme P. De Vuyst L.. (2007). *Lactobacillus crustorum* sp. nov., isolated from two traditional Belgian wheat sourdoughs. Int. J. Syst. Evol. Microbiol. 57, 1461–1467. 10.1099/ijs.0.64836-0 17625176

[B85] Scheirlinck I. van der Meulen R. van Schoor A. Vancanneyt M. De Vuyst L. Vandamme P.. (2008). Taxonomic structure and stability of the bacterial community in Belgian sourdough ecosystems as assessed by culture and population fingerprinting. Appl. Environ. Microbiol. 74, 2414–2423. 10.1128/AEM.02771-07 18310426 PMC2293155

[B86] Schmieder R. Edwards R. (2011). Quality control and preprocessing of metagenomic datasets. Bioinformatics 27, 863–864. 10.1093/bioinformatics/btr026 21278185 PMC3051327

[B87] Settanni L. (2017). Sourdough and cereal-based foods: traditional and innovative products, in: Starter Cultures in Food Production, eds Speranza B. Bevilacqua A. Corbo M. R. Sinigaglia M. (Chichester: John Wiley and Sons), 199–230.

[B88] Sieuwerts S. Bron P. A. Smid E. J. (2018). Mutually stimulating interactions between lactic acid bacteria and *Saccharomyces cerevisiae* in sourdough fermentation. Food Sci. Technol. 90, 201–206. 10.1016/j.lwt.2017.12.022

[B89] Spitaels F. Li L. Wieme A. D. Balzarini T. Cleenwerck I. van Landschoot A.. (2014a). *Acetobacter lambici* sp. nov., isolated from fermenting lambic beer. Int. J. Syst. Evol. Microbiol. 64, 1083–1089. 10.1099/ijs.0.057315-0 24363299

[B90] Spitaels F. Wieme A. D. Balzarini T. Cleenwerck I. van Landschoot A. De Vuyst L.. (2014b). *Gluconobacter cerevisiae* sp. nov., isolated from the brewery environment. Int. J. Syst. Evol. Microbiol. 64, 1134–1141. 10.1099/ijs.0.059311-0 24368694

[B91] Spitaels F. Wieme A. D. Janssens M. Aerts M. Daniel H.-M. van Landschoot A.. (2014c). The microbial diversity of traditional spontaneously fermented lambic beer. PLoS ONE 9:e95384. 10.1371/journal.pone.0095384 24748344 PMC3991685

[B92] Spitaels F. Wieme A. D. Janssens M. Aerts M. van Landschoot A. De Vuyst L.. (2015). The microbial diversity of an industrially produced lambic beer shares members of a traditionally produced one and reveals a core microbiota for lambic beer fermentation. Food Microbiol. 49, 23–32. 10.1016/j.fm.2015.01.008 25846912

[B93] Spitaels F. Wieme A. D. Snauwaert I. De Vuyst L. Vandamme P. (2017). Microbial ecology of traditional beer fermentations, in Brewing Microbiology: Current Research, Omics and Microbial Ecology, eds Bokulich N. Bamforth C. (Poole: Caister Academic Press), 179–196.

[B94] Succi M. Reale A. Andrighetto C. Lombardi A. Sorrentino E. Coppola R. (2003). Presence of yeasts in southern Italian sourdoughs from *Triticum aestivum* flour. FEMS Microbiol. Lett. 225, 143–148. 10.1016/S0378-1097(03)00500-7 12900033

[B95] Tatusova T. Ciufo S. Fedorov B. O'Neill K. Tolstoy I. (2014). RefSeq microbial genomes database: new representation and annotation strategy. Nucleic Acids Res. 42, D553–D559. 10.1093/nar/gkt1274 24316578 PMC3965038

[B96] Ua-Arak T. Jakob F. Vogel R. F. (2016). Characterization of growth and exopolysaccharide production of selected acetic acid bacteria in buckwheat sourdoughs. Int. J. Food Microbiol. 239, 103–112. 10.1016/j.ijfoodmicro.2016.04.009 27118673

[B97] Ua-Arak T. Jakob F. Vogel R. F. (2017). Influence of levan-producing acetic acid bacteria on buckwheat-sourdough breads. Food Microbiol. 65, 95–104. 10.1016/j.fm.2017.02.002 28400025

[B98] Van Kerrebroeck S. Maes D. De Vuyst L. (2017). Sourdoughs as a function of their species diversity and process conditions, a meta-analysis. Trends Food Sci. Technol. 68, 152–159. 10.1016/j.tifs.2017.08.016

[B99] Vassart G. Georges M. Monsieur R. Brocas H. Lequarre A. S. Christophe D. (1987). A sequence in M13 phage detects hypervariable minisatellites in human and animal DNA. Science. 235, 683–684. 10.1126/science.2880398 2880398

[B100] Verce M. De Vuyst L. Weckx S. (2019). Shotgun metagenomics of a water kefir fermentation ecosystem reveals a novel *Oenococcus* species. Front. Microbiol. 10:479. 10.3389/fmicb.2019.00479 30918501 PMC6424877

[B101] Verce M. De Vuyst L. Weckx S. (2020). The metagenome-assembled genome of *Candidatus* Oenococcus aquikefiri from water kefir represents the species *Oenococcus sicerae*. Food Microbiol. 88:103402. 10.1016/j.fm.2019.103402 31997765

[B102] Vernocchi P. Valmorri S. Dalai I. Torriani S. Gianotti A. Suzzi G. (2004). Characterization of the yeast population involved in the production of a typical Italian bread. J. Food Sci. 69, M182–M186. 10.1111/j.1365-2621.2004.tb13618.x

[B103] Versalovic J. Schneider M. De Bruijn F. J. Lupski J. R. (1994). Genomic fingerprinting of bacteria using repetitive sequence-based polymerase chain reaction. Methods Mol. Cell. Biol. 5, 25–40. 8750201

[B104] Vigentini I. Antoniani D. Roscini L. Comasio A. Galafassi S. Picozzi C.. (2014). *Candida milleri* species reveals intraspecific genetic and metabolic polymorphisms. Food Microbiol. 42, 72–81. 10.1016/j.fm.2014.02.011 24929720

[B105] Viiard E. Bessmeltseva M. Simm J. Talve T. Aaspõllu A. Paalme T.. (2016). Diversity and stability of lactic acid bacteria in rye sourdoughs of four bakeries with different propagation parameters. PLoS ONE 11:e0148325. 10.1371/journal.pone.0148325 26849134 PMC4743960

[B106] Vogel R. F. (2015). Microbial networks and metabolic fluxes in food fermentations, in Microbial Diversity - The Challenge of Complexity, eds Cardinali G. Corte L. Roscini L. Casella S. Cocolin L. Neviani E. (Firenze: Simtrea), 19–24.

[B107] Vogel R. F. Pavlovic M. Ehrmann M. A. Wiezer A. Liesegang H. Offschanka S.. (2011). Genomic analysis reveals *Lactobacillus sanfranciscensis* as stable element in traditional sourdoughs. Microb. Cell. Fact. 10:S6. 10.1186/1475-2859-10-S1-S6 21995419 PMC3231932

[B108] Wei T. Simko V. (2017). Corrplot: Visualization of a Correlation Matrix. R package version 0.84. Available online at: https://cran.r-project.org/web/packages/corrplot (accessed April 1, 2019).

[B109] White T. J. Bruns T. Lee S. Taylor J. W. (1990). Amplification and direct sequencing of fungal ribosomal RNA genes for phylogenetics, in PCR Protocols: A Guide to Methods and Applications, eds Innis M. A. Gelfand D. H. Sninsky J. J. White T. J. (New York, NY: Academic Press), 315–322.

[B110] Wickham H. (2009). ggplot2: Create Elegant Data Visualisations Using the Grammar of Graphics R Package Version 1.3.0. Available online at: https://cran.r-project.org/web/packages/ggplot2 (accessed April 20, 2020).

[B111] Wickham H. (2016). Scales: Scale Functions for Visualization. R package version 0.4.0. Available online at: https://cran.r-project.org/web/packages/scales (accessed June 3, 2020).

[B112] Wickham H. (2017). reshape2: Flexibly Reshape Data: A Reboot of the Reshape Package. R package version 1.4.3. Available online at: https://cran.r-project.org/web/packages/reshape2 (accessed April 1, 2019).

[B113] Wood D. E. Salzberg S. L. (2014). Kraken: ultrafast metagenomic sequence classification using exact alignments. Genome Biol. 15:R46. 10.1186/gb-2014-15-3-r46 24580807 PMC4053813

[B114] Zhang G. He G. (2013). Predominant bacteria diversity in Chinese traditional sourdough. J. Food Sci. 78, M1218–M1223. 10.1111/1750-3841.12193 23957410

[B115] Zheng J. Wittouck S. Salvetti E. Franz C. M. A. P. Harris H. M. B. Mattarelli P.. (2020). A taxonomic note on the genus *Lactobacillus*: description of 23 novel genera, emendeddescription of the genus *Lactobacillus* Beijerinck 1901, and union of *Lactobacillaceae* and *Leuconostocaceae*. Int. J. Syst. Evol. Microbiol. 70, 2782–2858. 10.1099/ijsem.0.004107 32293557

